# Phenotype-specific 25-hydroxyvitamin D and fat-soluble vitamin profiles in girls with pediatric endocrine disorders: a large cross-sectional study

**DOI:** 10.3389/fnut.2026.1924137

**Published:** 2026-08-28

**Authors:** Changzhen Li, Lei Xi, Jingjing Rao, Shiyong Deng, Ran Li, Lifang Feng, Feng Tang, Xiaomei Wang

**Affiliations:** 1 Department of Laboratory Medicine, Wuhan Children’s Hospital (Wuhan Maternal and Child Healthcare Hospital), Tongji Medical College, Huazhong University of Science & Technology, Wuhan, China; 2 Department of Clinical Laboratory Medicine, Haikou People’s Hospital, Affiliated Haikou Hospital, Xiangya School of Medicine, Central South University, Haikou, Hainan, China; 3 Department of Endocrinology, Wuhan Children’s Hospital (Wuhan Maternal and Child Healthcare Hospital), Tongji Medical College, Huazhong University of Science & Technology, Wuhan, China

**Keywords:** 25-hydroxyvitamin D, central precocious puberty, childhood obesity, cross-sectional study, fat-soluble vitamins, short stature, vitamin A, vitamin E

## Abstract

**Objective:**

To characterize serum vitamin A, total 25-hydroxyvitamin D [25(OH)D] and vitamin E across common pediatric endocrine disorders in girls and to test whether the profiles are phenotype-specific.

**Methods:**

In this single-center retrospective cross-sectional study, serum vitamin A, total 25(OH)D, and vitamin E were simultaneously measured by high-performance liquid chromatography in 4,111 girls aged 3–13 years, including healthy controls (*n* = 1,503), central precocious puberty (CPP; *n* = 1,146), short stature (*n* = 970), and obesity (*n* = 492). Age-adjusted effect sizes, logistic regression, restricted cubic spline analysis, and pre-specified sensitivity analyses were performed.

**Results:**

Each phenotype demonstrated a distinct fat-soluble vitamin profile. Obesity was characterized by higher vitamin A (adjusted Cohen’s *d* = +0.50) and lower 25(OH)D (*d* = −0.16), whereas CPP showed lower 25(OH)D (*d* = −0.26) and lower vitamin E (*d* = −0.25). In contrast, short stature was associated with higher 25(OH)D (*d* = +0.26). Vitamin D deficiency was more common in CPP (OR 1.43, 95% CI 1.20–1.70) but less common in short stature (OR 0.60, 95% CI 0.48–0.74). Restricted cubic spline analysis demonstrated a significant nonlinear inverse association between continuous 25(OH)D concentrations and CPP. The higher 25(OH)D concentrations observed in short stature persisted after excluding participants positive for the vitamin D2 supplementation marker.

**Conclusion:**

In girls, common pediatric endocrine disorders carry distinct, phenotype-specific fat-soluble vitamin profiles, with CPP and short stature diverging in opposite directions for vitamin D. Effect sizes were modest, and the short-stature elevation may substantially reflect unmeasured cholecalciferol supplementation; these group-level patterns therefore warrant cautious, biomarker-oriented interpretation.

## Introduction

Fat-soluble vitamins A, D, and E each play biological roles that intersect with childhood growth and pubertal development: vitamin D in skeletal mineralization and calcium homeostasis (1), vitamin A (retinol) in cell differentiation and the somatotropic axis (2), and vitamin E as the main lipid-soluble antioxidant (3). Although these micronutrients are routinely measured in pediatric endocrine practice, research has largely examined them individually, focusing on one vitamin and one phenotype at a time. It therefore remains unknown whether different pediatric endocrine disorders carry distinct, coordinated fat-soluble vitamin profiles: no large study has compared vitamins A, D and E simultaneously across multiple pediatric endocrine phenotypes, and the precocious end of the pubertal spectrum is essentially uncharacterized for vitamins A and E.

Beyond these classical functions, all three vitamins act as gene regulators rather than simple cofactors: 1,25-dihydroxyvitamin D signals through the vitamin D receptor (VDR), a ligand-activated transcription factor (4, 5) expressed in the human hypothalamus (6); retinoid and vitamin D signaling converge on shared nuclear-receptor machinery through the retinoid X receptor (RXR), the obligate heterodimeric partner of both the VDR and the retinoic acid receptor (5); and *α*-tocopherol regulates gene expression independently of radical scavenging (7). This shared regulatory biology is the rationale for measuring the three vitamins together rather than separately; the candidate mechanisms are examined in the Discussion.

The relationship between vitamin D and central precocious puberty (CPP) is already well characterized. Multiple meta-analyses agree that girls with CPP have lower circulating 25-hydroxyvitamin D [25(OH)D] than controls and that vitamin D deficiency is more common among them (8–10). Because this association has been repeatedly replicated and synthesized, re-examining vitamin D in CPP alone would add little; the open questions concern the other fat-soluble vitamins and other phenotypes. For vitamin A, the available evidence is markedly asymmetric. Studies have focused on the delayed end of the pubertal spectrum: children with constitutional delay of growth and puberty have lower serum vitamin A than peers (11), and supplementation with vitamin A and iron can advance growth and pubertal progression to a degree comparable with hormonal therapy (12). In healthy children, serum retinol and its transport protein increase with advancing pubertal stage (13, 14), suggesting that vitamin A availability is linked to sexual maturation. Whether retinol status is altered in CPP, however, has scarcely been examined. The relationship between vitamin E and precocious puberty is an even larger gap. Puberty is accompanied by measurable shifts in oxidant–antioxidant balance (15, 16), and metabolomic and lipidomic studies of girls with CPP consistently identify dysregulated lipid pathways (17, 18); as the principal lipid-soluble antioxidant, vitamin E is a plausible participant in this redox shift, yet it has not been systematically measured in children with CPP.

Obesity provides a useful directional anchor for interpreting any retinol findings. Retinol-binding protein 4 (RBP4), the circulating carrier of retinol, is consistently elevated in children with obesity and normalizes with weight loss (19, 20), and increases further with both adiposity and pubertal stage (19, 21). Conversely, the lipid-soluble antioxidant tocopherol tends to be lower in children with obesity and metabolic syndrome (16). These observations generate an *a priori* expectation: higher retinol and lower vitamin E in the obesity group, against which our data can be judged. Short stature serves as a specificity control. The vitamin D literature in short stature is mixed; using high-performance liquid chromatography–tandem mass spectrometry, short-stature children have been reported to have lower vitamin D than healthy controls (22). A difference in the short-stature group in the opposite direction would indicate that the vitamin findings are not produced by a single nonspecific consequence of clinical referral, although a supplementation-driven divergence would then still have to be distinguished from a phenotype-driven one.

To our knowledge, this is the first large-scale study to measure vitamins A, D and E simultaneously, on a single HPLC platform, across three pediatric endocrine phenotypes — CPP, short stature and obesity — and to test whether each disorder carries a phenotype-specific fat-soluble vitamin profile. Using a large clinical HPLC database from a tertiary children’s hospital, we (i) characterized the combined fat-soluble vitamin profiles of CPP, short stature, and obesity compared to healthy controls; (ii) tested whether these profiles differ by phenotype, using short stature as an opposite-direction control; and (iii) quantified the strength of these associations using age-adjusted effect sizes, with season and adiposity addressed in sensitivity analyses, to provide a more conservative benchmark than previous smaller studies.

## Methods

### Study design

This was a single-center, retrospective, cross-sectional study based on existing laboratory and electronic medical records at Wuhan Children’s Hospital (Wuhan Maternal and Child Healthcare Hospital), covering fat-soluble vitamin panels performed between July 2022 and April 2026, with one record retained per patient (the earliest available test). Reporting follows the STROBE statement for observational studies (23).

### Study population

**Figure 1:**
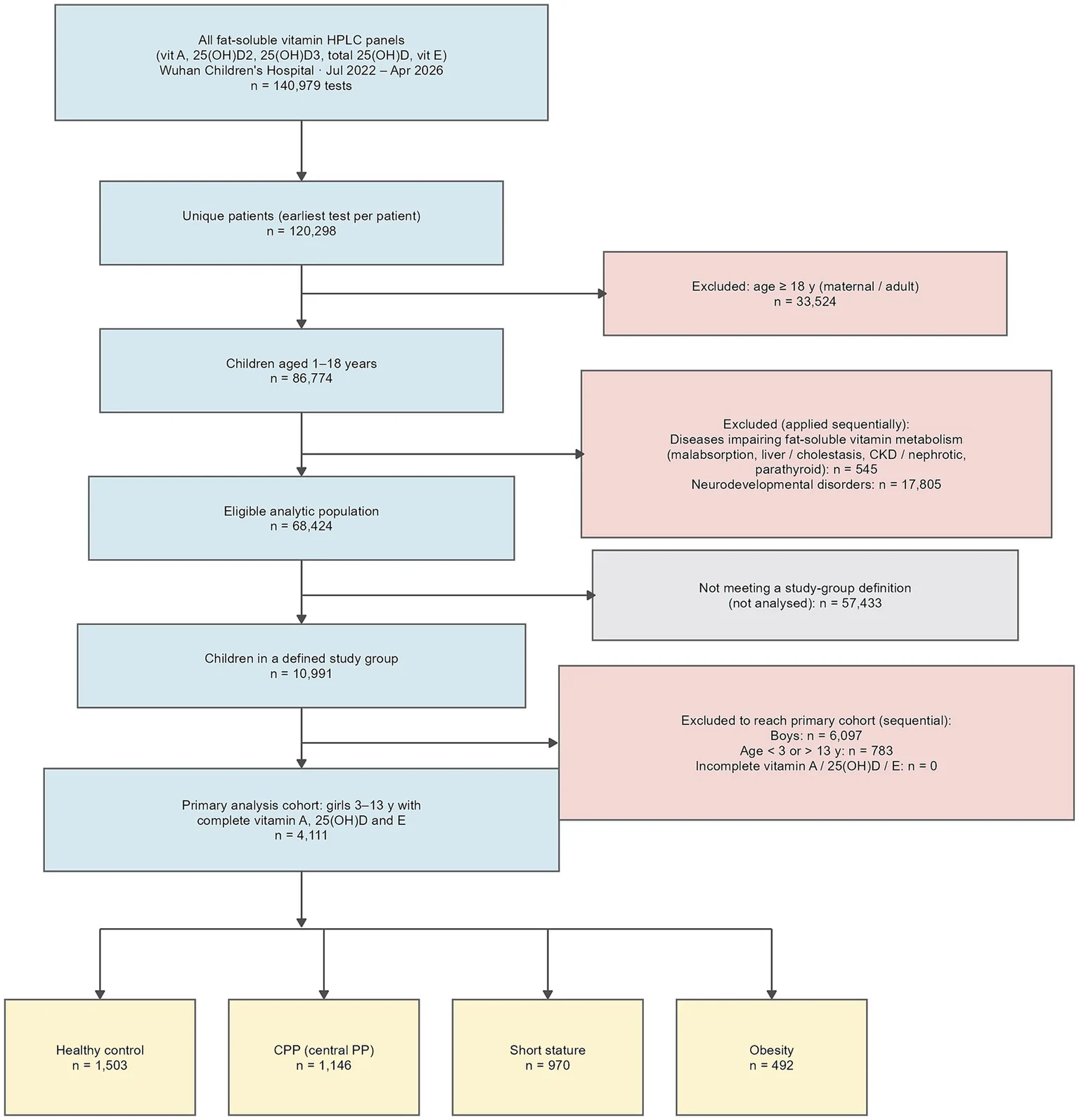
Flow of participants through the study. Derivation of the analysis cohort from the fat-soluble vitamin HPLC database at Wuhan Children’s Hospital (July 2022–April 2026). From 140,979 vitamin panels, one record per patient was retained (earliest test; 120,298 patients); children aged 1–18 years were kept (*n* = 86,774); and those with diseases impairing fat-soluble vitamin absorption or metabolism (intestinal malabsorption, chronic liver disease or cholestasis, chronic kidney disease or nephrotic syndrome, parathyroid disorders; *n* = 545) and neurodevelopmental disorders (*n* = 17,805, excluded for the reasons given in the Methods and characterized separately in our neurodevelopmental cohort) were excluded, leaving 68,424 eligible children. Of these, 10,991 met a study-group definition; the remainder did not meet any study-group definition and were not analyzed (*n* = 57,433). The primary analysis cohort comprised girls aged 3–13 years with complete serum vitamin A, 25(OH)D, and vitamin E (*n* = 4,111), forming four phenotype groups: healthy control (*n* = 1,503), Central Precocious Puberty (CPP; *n* = 1,146), short stature (*n* = 970), and obesity (*n* = 492).

Children were drawn from all patients for whom a fat-soluble vitamin panel was performed during the study period (Figure 1). Before grouping, the following were excluded sequentially: diseases known to impair fat-soluble vitamin absorption or metabolism (intestinal malabsorption, chronic liver disease or cholestasis, chronic kidney disease or nephrotic syndrome, and parathyroid disorders), and neurodevelopmental disorders. The latter were excluded on substantive grounds: selective or restrictive eating, reduced outdoor activity and consequently reduced cutaneous vitamin D synthesis, and, in children with epilepsy, enzyme-inducing antiseizure medication that accelerates 25(OH)D catabolism, are all independent determinants of fat-soluble vitamin status and would have confounded comparisons based on growth and pubertal phenotype. A secondary consideration was that these children are characterized separately in our companion study of a neurodevelopmental cohort (24), so the present cohort does not overlap with that study.

Children were assigned to mutually exclusive phenotype groups based on the clinical diagnosis recorded at the testing encounter. Four groups were analyzed: (i) healthy control, children who attended the outpatient clinic for routine health examination and had no documented diagnosis of any growth, pubertal, endocrine, metabolic, neurodevelopmental, chronic medical, or acute illness condition at the time of the visit in their outpatient medical records; (ii) central precocious puberty (CPP), a recorded diagnosis of central precocious puberty (development of secondary sexual characteristics before 8 years in girls, of central/GnRH-dependent origin) (25, 26); (iii) short stature, a recorded diagnosis of short stature or growth retardation (height ≤ −2 SD for age and sex) (27); and (iv) obesity, a recorded diagnosis of obesity (body mass index at or above the age- and sex-specific cutoff) (28).

The reference standards applied in routine clinical practice were as follows. The reference criterion for short stature was height ≤ −2 SD for age and sex, the diagnostic threshold of the international consensus statement (27), evaluated against the LMS-based standardized growth charts for Chinese children and adolescents aged 0–18 years (29); the criterion for obesity was body mass index at or above the age- and sex-specific 95th percentile of the Working Group on Obesity in China (WGOC) reference (28). Central precocious puberty was diagnosed by pediatric endocrinologists according to the prevailing consensus guidelines (25, 26, 30), which in our institution require biochemical confirmation of central origin by gonadotropin-releasing hormone (GnRH) stimulation testing, with a peak luteinizing hormone (LH) ≥ 5 IU/L and a peak LH/follicle-stimulating hormone ratio ≥ 0.6, measured by chemiluminescent immunoassay. Group assignment was based on the diagnosis recorded by the attending clinician applying these standards, and not on our own re-application of the reference standards to raw measurements; because the study was retrospective, we could not verify from the electronic record that a documented stimulation test was available for every case. As an internal check on the recorded labels, measured height and weight retrieved for this revision were broadly concordant with them (Supplementary Table S16B): median height-for-age Z in the short-stature group was −2.21 (IQR − 2.46, −1.94) versus +0.19 (−0.52, +0.83) in controls (*p* < 0.0001), with 68.0% of the short-stature group at or below the −2 SD diagnostic threshold and 82.0% at or below the 3rd centile, compared with 1.3% of controls; and 91.3% of the obesity group were at or above the 95th centile of the national BMI-for-age reference, compared with 10.8% of controls and 3.1% of the short-stature group.

For healthy controls, classification was based on the absence of any diagnostic code indicative of a growth, pubertal, endocrine, metabolic, neurodevelopmental, or other relevant medical condition in the electronic medical record at the time of the routine-health-examination visit; no active clinical re-screening was performed, consistent with the retrospective design. When a child had more than one qualifying diagnosis, group assignment followed a pre-specified clinical-severity hierarchy (CPP > short stature > obesity): central precocious puberty, as activation of the hypothalamic–pituitary–gonadal axis, was treated as the dominant diagnosis when present, while obesity (an upstream risk factor for, rather than a phenotype parallel to, precocious puberty and short stature) was assigned lowest priority, so a child with both CPP and obesity was classified as CPP (31). Children meeting none of these definitions were classified as “not meeting a study-group definition” and were not analyzed. This applied to 57,433 of the 68,424 eligible children (84%; Figure 1). Both cases and controls were therefore drawn from the subset of children who underwent fat-soluble vitamin testing during a hospital visit, so the control group constitutes a tested reference subset rather than a population-representative sample, and all between-group comparisons reported here should be read as relative to this tested subset rather than to the general pediatric population. A broader definition of precocious puberty (including peripheral precocity and affected boys) was used only in sensitivity analysis.

The primary analysis was limited to girls aged 3–13 years with complete serum measurements of vitamin A, total 25(OH)D, and vitamin E. This window was specified *a priori*. The lower bound of 3 years excludes infancy and early toddlerhood, when guideline-recommended prophylactic vitamin D supplementation is nearly universal in Chinese children and systematically raises circulating 25(OH)D (32–35), so that including this age range would confound the vitamin D profile and the vitamin D2 supplementation marker. The upper bound of 13 years corresponds to the upper limit of normal pubertal onset in girls (36, 37), defining the developmental interval within which CPP (onset of secondary sexual characteristics before 8 years (25, 26)) and short stature are clinically defined and mutually comparable. The cohort was restricted to girls because CPP is strongly female-predominant (>90% of cases) (25); a single-sex cohort maximizes comparability across the phenotype groups and avoids unstable estimates from the few affected boys.

### Laboratory measurements

Serum retinol (vitamin A), 25-hydroxyvitamin D2 [25(OH)D2], 25-hydroxyvitamin D3 [25(OH)D3], and *α*-tocopherol (vitamin E) were quantified simultaneously from a single sample by high-performance liquid chromatography (HPLC) on the same clinical analyzer throughout the study period in the accredited clinical laboratory of Wuhan Children’s Hospital. Total 25(OH)D was calculated as the sum of 25(OH)D2 and 25(OH)D3. All concentrations are expressed in ng/mL. The laboratory participates in regular internal and external quality-control programs. Daily calibration used certified reference materials, internal quality-control samples were analyzed with each batch, and intra-assay and inter-assay coefficients of variation were less than 5% for all vitamin measurements. Vitamin status was classified using pre-specified thresholds: vitamin D deficiency, total 25(OH)D ≤ 20 ng/mL; insufficiency, >20 to 30 ng/mL; sufficiency, >30 ng/mL (38); vitamin A deficiency, <300 ng/mL; sufficiency, 300–800 ng/mL; excess, >800 ng/mL; vitamin E deficiency, <5168.6 ng/mL; sufficiency, 5168.6–20,000 ng/mL; excess, >20,000 ng/mL (clinical laboratory reference intervals). Because cutaneous synthesis and most dietary and supplemental sources other than ergocalciferol yield cholecalciferol (D3), a 25(OH)D2:25(OH)D3 ratio greater than 0.2 was used as a pragmatic, study-defined marker of vitamin D2 (ergocalciferol) supplementation, rather than a formally validated diagnostic value (1). Because no consensus cut-off exists for this ratio, the entire supplementation analysis was repeated *post hoc* across ratios of 0.10, 0.15, 0.20 and 0.30 (Supplementary Table S15).

### Statistical analysis

Continuous variables are summarized as median (interquartile range), and categorical variables as n (%); unadjusted between-group comparisons used the Kruskal–Wallis test and the χ^2^ test, respectively.

For each vitamin, the primary measure of effect was the age-adjusted standardized mean difference versus healthy controls (adjusted Cohen’s d), obtained as the group coefficient from a linear model fitted to the vitamin standardized to unit SD with age as a covariate. Deficiency and excess were modeled by age-adjusted logistic regression and summarized as odds ratios (OR) with profile-likelihood 95% confidence intervals. Specificity of the profiles was assessed by a direct, age-adjusted contrast of CPP versus short stature and by a full pairwise between-group comparison matrix (Kruskal–Wallis followed by pairwise Wilcoxon rank-sum tests). The shape of the association between continuous total 25(OH)D and phenotype was modeled with restricted cubic splines (four knots placed at the 5th, 35th, 65th, and 95th percentiles) (39).

The role of adiposity was examined in three ways. First, the CPP vitamin D deficiency association was stratified by obesity status. Second, for vitamin D deficiency we fitted sequential models with increasing adjustment: M1, unadjusted; M2, plus age; and M3, plus age, season, and obesity. Third, in a *post hoc* analysis added during the previous revision, adiposity was addressed as a continuous rather than a dichotomous covariate: all recorded height and weight measurements for the analytic cohort were retrieved from the electronic medical record and linked to the laboratory file by specimen barcode (4,111 of 4,111 matched). Weight was recorded for 3,548 girls (86.3%) and height for 992 (24.1%), permitting BMI to be computed for 980 (23.8%). Ten recorded weights were set to missing by a pre-specified plausibility screen (seven exceeded 100 kg with no height recorded and were consistent with a height entered in the weight field; three had a weight-for-age Z below −6, the WHO lower limit of biological plausibility), leaving 3,538 analyzable weights (86.1%); none of the ten carried a height, so the complete-anthropometry subgroup was unaffected. Weight-for-age Z, BMI-for-age Z and height-for-age Z were derived by the LMS method against the national growth references for Chinese children and adolescents aged 0–18 years (29, 40), using the L, M and S parameters published for the identical reference sample by Zong and Li (41) and interpolated to exact age in months with a monotone cubic spline. Continuous adiposity was therefore modeled at two levels: weight-for-age Z in the weight-based set, and measured BMI, entered both as the raw continuous value and as BMI-for-age Z, in the complete-anthropometry subgroup. Every adiposity-adjusted model was accompanied by an age-only model fitted in the identical subset, so that the effect of adjustment could be distinguished from the effect of sample restriction, and the internal validity of both subsets was tested formally with phenotype by data-availability interaction terms (Supplementary Table S16). The relative attenuation from the age-only to the adiposity-adjusted model, rather than the absolute adjusted estimate, was treated as the interpreted quantity.

Pre-specified sensitivity analyses included alternative age windows (2–14, 4–12, 2–12, and 4–14 years); the broad precocious-puberty definition including boys; sex-stratified profiles and sex-by-season strata; and the vitamin D2-supplementation analysis. To assess whether the primary effect sizes were sensitive to the functional form of age adjustment, we also re-estimated all primary models replacing the linear age term with a restricted cubic spline for age (four knots at the 5th, 35th, 65th, and 95th percentiles). To address multiplicity, *p* values for the primary analyses were additionally adjusted using the Benjamini–Hochberg false discovery rate (42), and statistical significance is indicated at four levels (*p* < 0.05, <0.01, <0.001, and <0.0001). Given the large sample size, interpretation emphasizes effect-size magnitude rather than statistical significance alone.

As a *post hoc* internal validation, we assessed the stability and discriminative accuracy of the primary vitamin profiles using (i) 2,000-iteration bootstrap resampling to obtain bias-corrected 95% confidence intervals for each age-adjusted Cohen’s d and each deficiency odds ratio; (ii) repeated 10 × 10-fold stratified cross-validation of logistic regression models (age plus all three vitamins) to estimate the area under the receiver operating characteristic curve (AUC) and Brier score for each phenotype versus healthy controls, as well as an age-only model fitted in the same folds to estimate the incremental discrimination (ΔAUC) contributed by the three vitamins beyond age; and (iii) leave-one-season-out cross-validation to verify that the profiles were not driven by seasonal sampling artifacts. Internal validation results are reported in Supplementary Table S12. Two exploratory multivariable analyses characterized the joint structure of the fat-soluble vitamins: pairwise Pearson correlations among the measured analytes, and unsupervised K-means clustering of the standardized concentrations of the four directly measured analytes (vitamin A, 25(OH)D2, 25(OH)D3, and vitamin E). Total 25(OH)D was excluded from clustering as the arithmetic sum of 25(OH)D2 and 25(OH)D3 to avoid exact collinearity; the number of clusters was selected by the elbow criterion and the mean silhouette width, and the distribution of clusters across phenotype groups was tested with the χ^2^ test. Because the resulting cluster separation was weak (mean silhouette ≈0.25), this analysis is presented as a hypothesis-free check rather than as evidence of distinct metabolic subtypes. All tests were two-sided. Analyses were performed in R (version 4.3.0; R Foundation for Statistical Computing, Vienna, Austria), with restricted cubic splines fitted using the rms package.

## Results

### Study population

The HPLC database included 4,111 girls aged 3–13 years with complete serum vitamin A, total 25(OH)D, and vitamin E data who met one of four phenotype definitions (healthy control, *n* = 1,503; CPP, *n* = 1,146; short stature, *n* = 970; obesity, *n* = 492; derivation in Figure 1). Baseline characteristics are presented in Table 1. The groups differed in age (medians 6–9 years). Overall, 29% of girls were vitamin D deficient (25(OH)D ≤ 20 ng/mL), and an additional 43% were insufficient (20–30 ng/mL). Median 25(OH)D was lower in CPP and obesity (both 22 ng/mL) than in healthy controls (27 ng/mL) and was highest in short stature (28 ng/mL), while serum vitamin A was highest in obesity (390 ng/mL vs. approximately 350 ng/mL in the other groups) (Table 1).

**Table 1:** Baseline characteristics and serum fat-soluble vitamins by phenotype group (girls 3–13 y).

Characteristic	Overall *N* = 4,111^1^	Control *N* = 1,503^1^	CPP *N* = 1,146^1^	Short stature *N* = 970^1^	Obesity *N* = 492^1^	*p*-value^2^
Age, years	7.8 (5.8, 9.3)	6.5 (4.3, 9.1)	8.2 (7.4, 9.3)	6.6 (5.0, 9.3)	8.8 (7.6, 10.2)	<0.001
Season						<0.001
Spring	1,032 (25%)	375 (25%)	282 (25%)	256 (26%)	119 (24%)	
Summer	1,405 (34%)	580 (39%)	316 (28%)	337 (35%)	172 (35%)	
Autumn	893 (22%)	278 (18%)	322 (28%)	191 (20%)	102 (21%)	
Winter	781 (19%)	270 (18%)	226 (20%)	186 (19%)	99 (20%)	
Vitamin A, ng/mL	359.6 (307.3, 415.3)	353.0 (301.5, 405.3)	358.8 (305.8, 417.1)	355.1 (306.3, 406.5)	390.2 (340.2, 458.8)	<0.001
25(OH)D total, ng/mL	24.9 (19.1, 30.9)	26.6 (20.2, 33.4)	22.3 (16.9, 26.9)	27.9 (22.2, 34.2)	21.6 (17.1, 27.0)	<0.001
Vitamin E, ng/mL	8,968.7 (7,573.7, 10,726.3)	9,295.9 (7,837.6, 11,059.6)	8,454.2 (7,207.5, 10,032.9)	9,324.3 (7,837.2, 11,098.4)	8,640.9 (7,334.9, 10,248.7)	<0.001
Vitamin D status						<0.001
Deficient	1,187 (29%)	369 (25%)	445 (39%)	176 (18%)	197 (40%)	
Insufficient	1,772 (43%)	599 (40%)	537 (47%)	407 (42%)	229 (47%)	
Sufficient	1,152 (28%)	535 (36%)	164 (14%)	387 (40%)	66 (13%)	
Vitamin A status						<0.001
Deficient	881 (21%)	370 (25%)	257 (22%)	201 (21%)	53 (11%)	
Sufficient	3,228 (79%)	1,133 (75%)	888 (77%)	769 (79%)	438 (89%)	
Excess	2 (<0.1%)	0 (0%)	1 (<0.1%)	0 (0%)	1 (0.2%)	
Vitamin E status						0.015
Deficient	52 (1.3%)	20 (1.3%)	21 (1.8%)	4 (0.4%)	7 (1.4%)	
Sufficient	4,051 (99%)	1,482 (99%)	1,124 (98%)	963 (99%)	482 (98%)	
Excess	8 (0.2%)	1 (<0.1%)	1 (<0.1%)	3 (0.3%)	3 (0.6%)	

^1^Median (Q1, Q3); n (%), ^2^Kruskal-Wallis rank sum test; Pearson’s Chi-squared test; Continuous variables, median (IQR); categorical, n (%). *P*-values: Kruskal-Wallis (continuous) and chi-square (categorical). Healthy control is the reference. Cut-offs (ng/mL): vitamin D deficiency 25(OH)D < =20; vitamin A deficiency <300, excess >800; vitamin E deficiency <5168.6, excess >20000.

### Fat-soluble vitamin profiles across phenotypes

**Figure 2:**
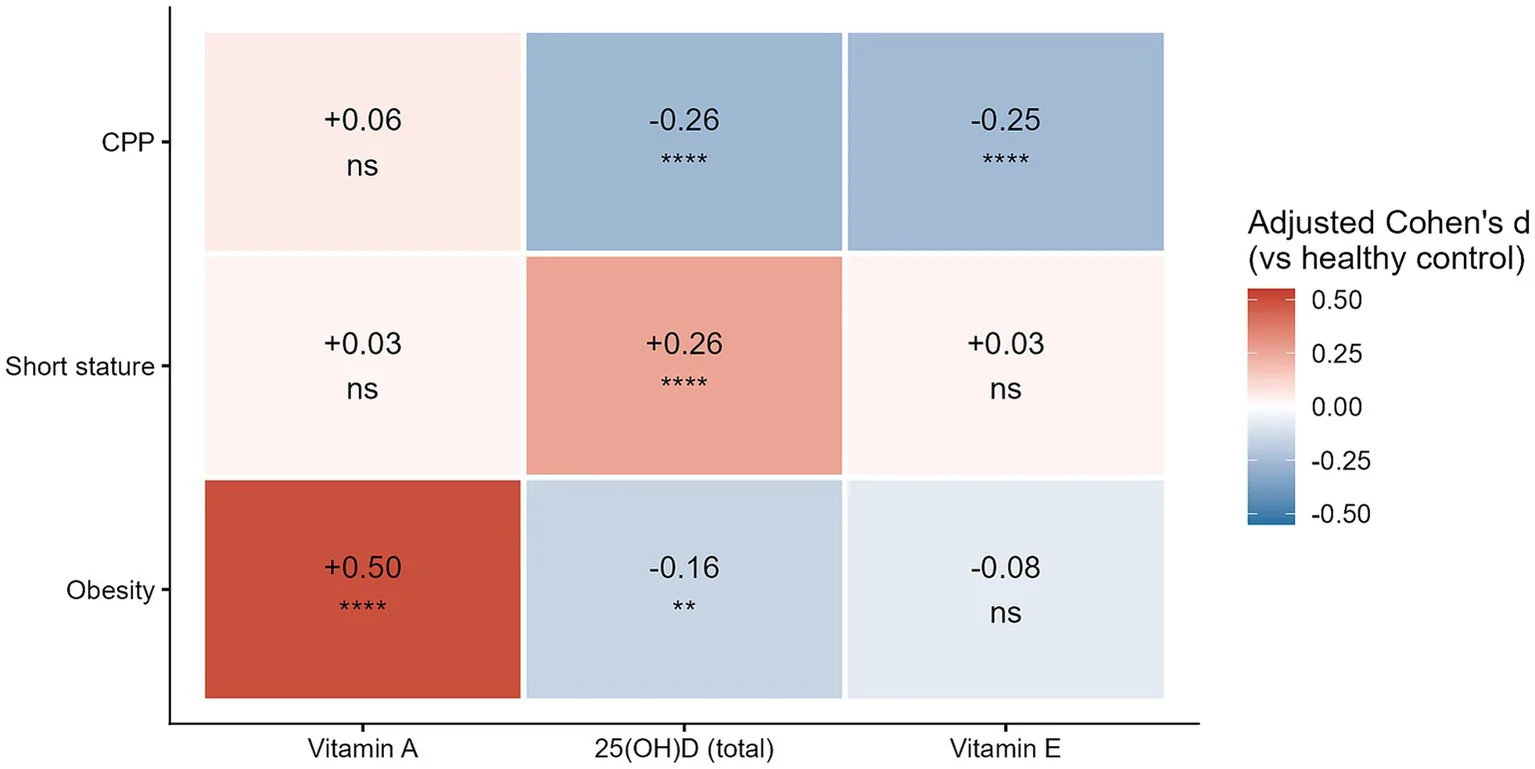
Fat-soluble vitamin profiles differ across pediatric endocrine phenotypes in girls. Age-adjusted standardized mean differences (Cohen’s *d*) in serum vitamin A, total 25-hydroxyvitamin D [25(OH)D], and vitamin E for each phenotype group relative to healthy controls, among girls aged 3–13 years with complete measurements of all three vitamins (healthy control, *n* = 1,503; central precocious puberty [CPP], *n* = 1,146; short stature, *n* = 970; obesity, *n* = 492). Each *d* was estimated as the group coefficient from a linear model fitted to the SD-standardized vitamin with age as a covariate; healthy controls are the reference (*d* = 0). Cell color encodes the direction and magnitude of *d* on a symmetric diverging scale (blue, lower than controls; red, higher than controls; range −0.55 to +0.55). Asterisks denote two-sided *p*-values adjusted for the false discovery rate (Benjamini–Hochberg) across the nine tests shown: *p < 0.05, **p < 0.01, ***p < 0.001, ****p < 0.0001; ns, not significant. The three phenotypes show distinct profiles; in particular, 25(OH)D is lower in CPP (*d* = −0.26) but higher in short stature (*d* = +0.26); the short-stature elevation may, however, substantially reflect differential vitamin D supplementation (see Discussion). Vitamin A is markedly higher only in obesity (*d* = +0.50).

**Figure 3:**
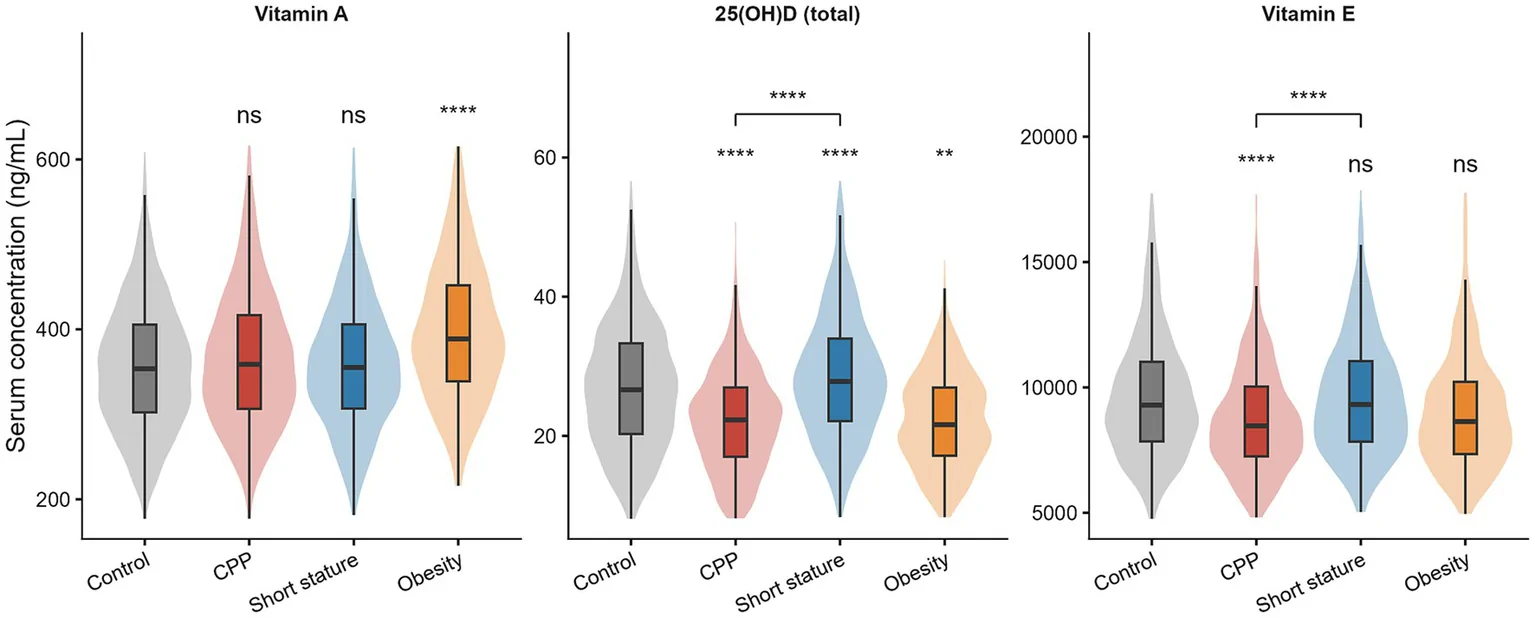
Distributions of serum fat-soluble vitamins by phenotype group. Violin (distribution) and box (median and interquartile range) plots of serum vitamin A, total 25-hydroxyvitamin D [25(OH)D], and vitamin E in girls aged 3–13 years with complete measurements, by phenotype group (healthy control, *n* = 1,503; central precocious puberty [CPP], *n* = 1,146; short stature, *n* = 970; obesity, *n* = 492). Each panel has an independent y-axis; for display, values were truncated to the 0.5th–99.5th percentile per vitamin, which does not affect medians or quartiles. Markers above each group denote the comparison with healthy controls using the same age-adjusted, false-discovery-rate–corrected (Benjamini–Hochberg) tests reported in Figure 2 and Table 2; that is, the group coefficient from an age-adjusted model, not an unadjusted comparison of the raw distributions shown: *p < 0.05, **p < 0.01, ***p < 0.001, ****p < 0.0001; ns, not significant. The bracket on the 25(OH)D and vitamin E panels denotes the direct CPP-versus-short-stature comparison (pairwise Wilcoxon with Benjamini–Hochberg correction, Supplementary Table S7; concordant with the age-adjusted contrast in Supplementary Table S6): ****p < 0.0001. In the 25(OH)D panel, concentrations are lower in CPP and obesity and higher in short stature relative to controls. Vitamin A is distinctly higher only in obesity.

Each phenotype exhibited a distinct, age-adjusted fat-soluble vitamin profile compared to healthy controls (Figure 2 and Table 2; distributions in Figure 3). Obesity was marked by significantly higher vitamin A (adjusted Cohen’s *d* = +0.50; *p* < 0.0001) and lower 25(OH)D (*d* = −0.16; *p* < 0.01). CPP showed a different pattern, with lower 25(OH)D (*d* = −0.26; *p* < 0.0001) and lower vitamin E (*d* = −0.25; *p* < 0.0001), and no significant increase in vitamin A (*d* = +0.06; not significant after false-discovery-rate correction). Consistent with this, the bootstrap 95% confidence interval included zero (−0.02 to +0.13), and the direction was consistent in only 7 of 10 cross-validation folds (Supplementary Table S12). In contrast, short stature was associated with higher 25(OH)D than controls (*d* = +0.26; *p* < 0.0001), while vitamin A and vitamin E remained unchanged. The magnitude of each individual effect was small to moderate (|d| ≤ 0.50).

**Table 2:** Adjusted fat-soluble vitamin profiles vs. healthy control (girls 3–13 y).

**(A)** Adjusted standardized difference, Cohen’s *d* (95% CI)			
Vitamin	CPP vs control	Short stature vs control	Obesity vs control
Vitamin A	+0.06 (−0.02, 0.13)	+0.03 (−0.05, 0.11)	+0.50 (0.39, 0.60)
p (FDR p)	0.158 (0.203)	0.461 (0.461)	<0.001 (<0.001)
25(OH)D (total)	−0.26 (−0.33, −0.20)	+0.26 (0.19, 0.33)	−0.16 (−0.25, −0.07)
p (FDR p)	<0.001 (<0.001)	<0.001 (<0.001)	<0.001 (0.001)
Vitamin E	−0.25 (−0.33, −0.18)	+0.03 (−0.04, 0.11)	−0.08 (−0.18, 0.03)
p (FDR p)	<0.001 (<0.001)	0.392 (0.441)	0.148 (0.203)

(B) Adjusted odds ratio of deficiency / excess (95% CI)			
**Outcome**	**CPP vs control**	**Short stature vs control**	**Obesity vs control**
Vitamin D deficiency (<=20)	1.43 (1.20–1.70)	0.60 (0.48–0.74)	1.23 (0.98–1.55)
p (FDR p)	<0.001 (<0.001)	<0.001 (<0.001)	0.076 (0.115)
Vitamin A deficiency (<300)	0.95 (0.79–1.15)	0.81 (0.67–0.99)	0.41 (0.30–0.56)
p (FDR p)	0.632 (0.702)	0.038 (0.068)	<0.001 (<0.001)
Vitamin E excess (>20000)	1.75 (0.07–47.60)	4.96 (0.63–100.63)	13.17 (1.46–293.18)
p (FDR p)	0.702 (0.702)	0.167 (0.215)	0.037 (0.068)

All models adjusted for age. **(A)** Each vitamin standardized to SD = 1; the group coefficient is the adjusted Cohen’s *d* vs. control. **(B)** Logistic-regression odds ratios vs. control (profile-likelihood CIs). Benjamini-Hochberg FDR-adjusted p across the 9 tests in each panel in parentheses (primary significance criterion). The vitamin E excess category comprises very few events across the cohort (*n* = 8); its odds ratios are therefore statistically unstable (note the wide confidence intervals) and are not interpreted.

### Vitamin D in CPP and short stature: deficiency odds and dose–response

**Figure 4:**
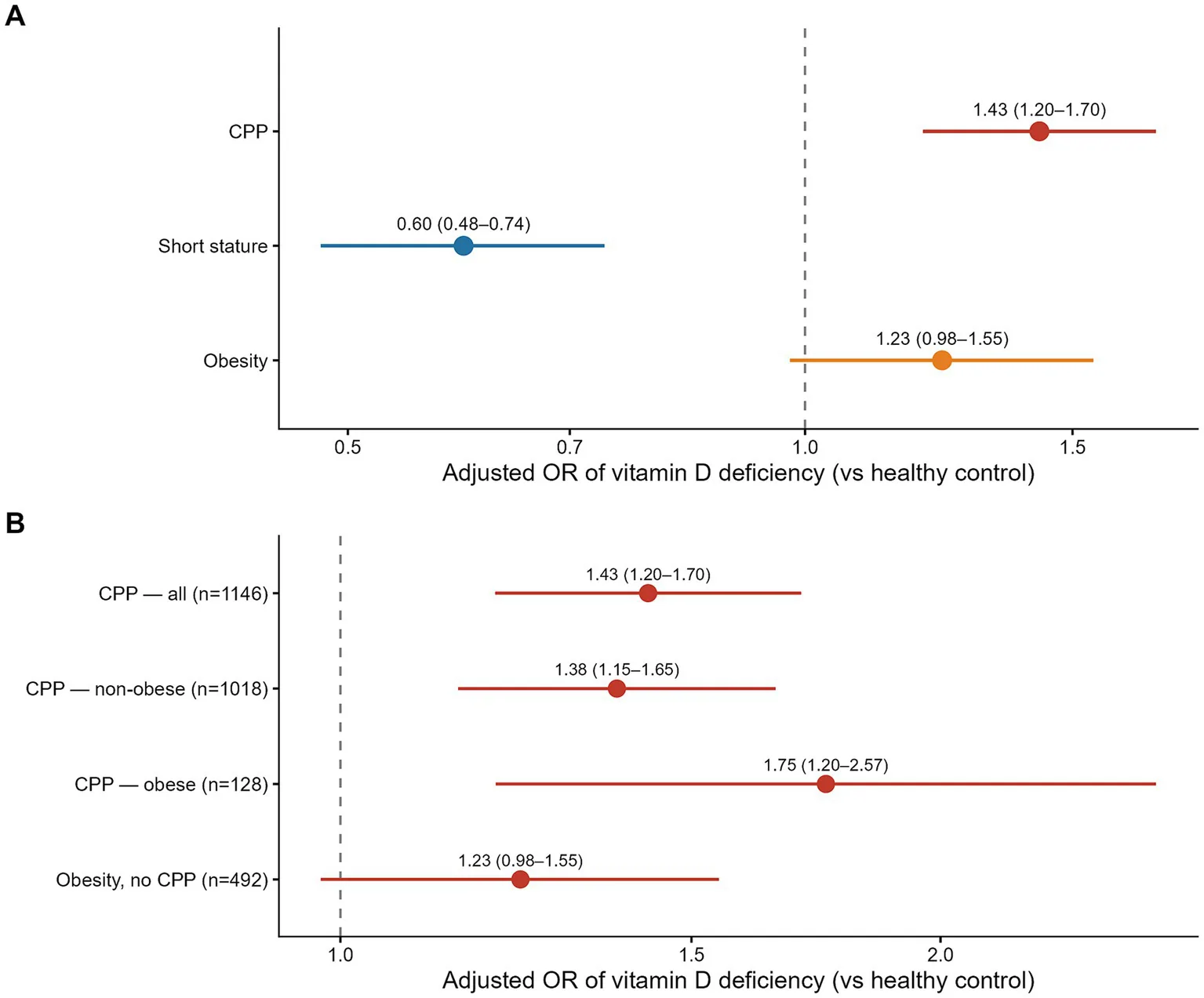
Vitamin D deficiency risk by phenotype **(A)** and by obesity stratum **(B)**. Adjusted odds ratios (*OR*) of vitamin D deficiency (total 25(OH)D ≤ 20 ng/mL) with 95% confidence intervals, plotted on a log scale; the dashed line marks *OR* = 1 (healthy controls, reference). **(A)** Between-phenotype contrast. Each phenotype group versus healthy controls, age-adjusted, from a single logistic model including all phenotype groups (the same model as Table 2): CPP 1.43 (1.20–1.70), short stature 0.60 (0.48–0.74), obesity 1.23 (0.98–1.55). Odds of deficiency were higher in CPP and lower in short stature than in controls; the lower odds in short stature are consistent with their higher 25(OH)D and may substantially reflect differential vitamin D supplementation (see Discussion). **(B)** Obesity-stratified estimates. Age-adjusted *OR* of vitamin D deficiency for CPP overall and within obesity strata, each versus the same healthy controls (*n* per row). The CPP-overall and obesity-only rows are from the all-groups model (as in **A**); the within-CPP obesity strata are stratum-specific models. The elevated odds in CPP persist in the non-obese stratum (1.38, 1.15–1.65), while obesity without CPP is not significantly associated (1.23, 0.98–1.55).

**Figure 5:**
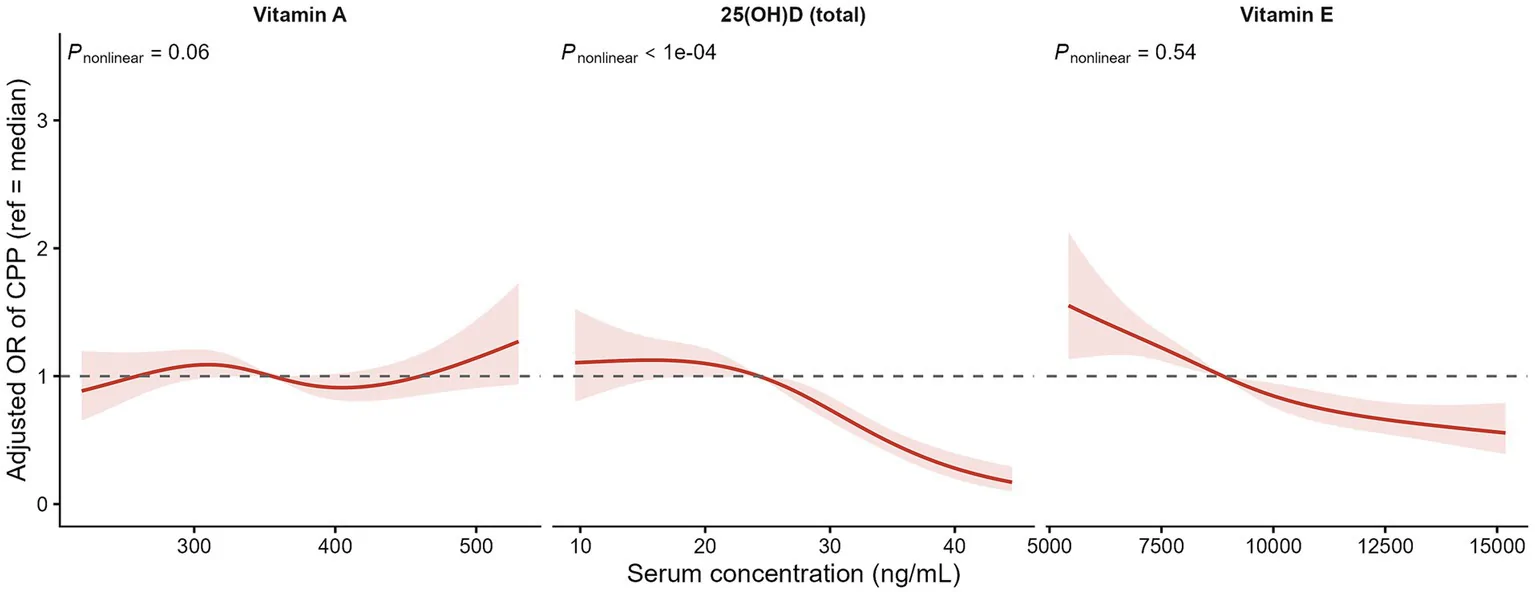
Restricted cubic spline association between each fat-soluble vitamin and the odds of central precocious puberty. Adjusted odds ratios (OR) of central precocious puberty (CPP) versus healthy control as a function of serum vitamin A, total 25-hydroxyvitamin D [25(OH)D], and vitamin E, among girls aged 3–13 years (CPP, *n* = 1,146; control, *n* = 1,503). Each curve is from a logistic regression modeling the vitamin with a restricted cubic spline (4 knots at the 5th, 35th, 65th, and 95th percentiles) and adjusting for age; the OR is expressed relative to the cohort median of each vitamin (reference, OR = 1, dashed line), and shaded bands are 95% confidence intervals. To avoid unreliable linear extrapolation beyond the boundary knots, curves are displayed over the 2.5th–97.5th percentile of each vitamin. *p*-values test departure from linearity (nonlinear spline terms). 25(OH)D shows a significant nonlinear inverse association (*P*nonlinear < 0.0001): The odds of CPP decline as 25(OH)D rises, with an inflection near 25–30 ng/mL. Vitamin E shows a graded inverse association without significant nonlinearity (*P*nonlinear = 0.54), and vitamin A shows no clear graded association (*P*nonlinear = 0.06).

The age-adjusted odds of vitamin D deficiency compared to healthy controls were increased in CPP (OR 1.43; 95% CI 1.20–1.70), decreased in short stature (OR 0.60; 0.48–0.74), and not significantly altered in obesity (OR 1.23; 0.98–1.55) (Figure 4A and Table 2). In the direct age-adjusted comparison with short stature, girls with CPP had lower 25(OH)D (*d* = −0.54; *p* < 0.0001) and lower vitamin E (*d* = −0.33; *p* < 0.0001), no difference in vitamin A, and higher odds of vitamin D deficiency (OR 2.40; 1.95–2.97) (Figure 3, 25(OH)D and vitamin E panels; Supplementary Table S6). The complete pairwise between-group matrix is provided in Supplementary Table S7. Restricted cubic spline models showed a significant nonlinear inverse association between continuous 25(OH)D and the odds of CPP (P for nonlinearity < 0.0001): the odds of CPP remained relatively flat at lower concentrations and then declined more steeply as 25(OH)D increased, with an inflection near 25–30 ng/mL (Figure 5). In 10-fold cross-validation, adding polynomial terms for 25(OH)D did not improve out-of-sample discrimination compared to a linear model (Supplementary Table S12). Vitamin E showed a graded, approximately monotonic inverse association without significant nonlinearity (*p* = 0.54), whereas vitamin A showed no clear graded association (*p* = 0.06).

### Adjustment for adiposity

We next tested whether the CPP association was confounded by adiposity. The elevated odds of vitamin D deficiency in CPP persisted in the non-obese group (OR 1.38; 1.15–1.65) and were even higher in the obese group (OR 1.75; 1.20–2.57), whereas obesity without CPP remained non-significant (Figure 4B and Supplementary Table S5). Sequential adjustment attenuated the CPP estimate from the crude model (M1, OR 1.95) mainly through age (M2, OR 1.42), with little further change after adding season and obesity (M3, OR 1.33; Supplementary Table S1). Adding continuous adiposity to the primary models attenuated but did not abolish the phenotype contrasts (Supplementary Table S16). Among girls with an analyzable weight (*n* = 3,538), the odds of vitamin D deficiency in CPP fell from 1.61 to 1.36 after adjustment for weight-for-age Z, with parallel attenuation of the continuous effects; in the subgroup with complete anthropometry (*n* = 980), the profiles were likewise preserved after adjustment for BMI-for-age Z, and the direct CPP-versus-short-stature divergence persisted (deficiency OR 2.02, 1.25–3.33). Adiposity itself was associated with the vitamins in the expected directions, and the phenotype contrasts persisted in the presence of this adiposity gradient (Supplementary Table S16). Girls in whom BMI could be computed differed from those in whom it could not in absolute vitamin concentrations but not in the phenotype contrasts (all phenotype × BMI-availability interactions *p* ≥ 0.12); the CPP × weight-availability interaction was significant, consistent with differential recording of weight across study years and ages, and attenuation was accordingly read against the age-only model fitted in the identical subset (Supplementary Table S16C).

### Sensitivity analyses and a vitamin D2 supplementation marker in short stature

**Figure 6:**
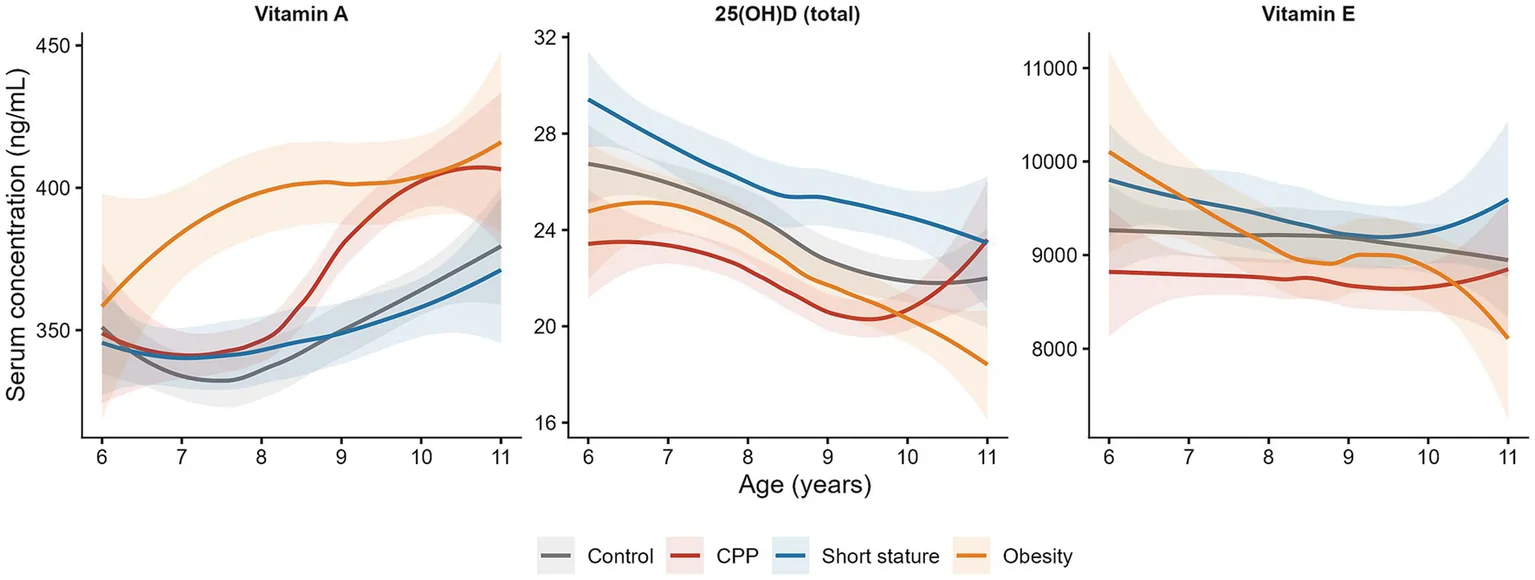
Age trajectories of serum fat-soluble vitamins by phenotype group. Locally weighted regression (LOESS, span 0.9) smooths of serum vitamin A, total 25-hydroxyvitamin D [25(OH)D], and vitamin E against age, by phenotype group, among girls aged 3–13 years with complete measurements (healthy control, *n* = 1,503; CPP, *n* = 1,146; short stature, *n* = 970; obesity, *n* = 492). To compare trajectories on equal footing, all curves are restricted to a common age window of 6–11 years, within which every group is well represented (*n* = 752/1,033/486/400 for control, CPP, short stature, and obesity); this avoids unstable extrapolation in the sparsely sampled tails (CPP and obesity contribute few children below ~6 years). Shaded bands are 95% confidence intervals. These are unadjusted trajectories shown for descriptive purposes; comparisons adjusted for age and multiplicity are in Figure 2 and Table 2 (e.g., the rise in vitamin A at older ages reflects the normal age-related increase in retinol rather than a phenotype effect). 25(OH)D declines with age in all groups, while the between-group ordering is broadly stable (short stature highest; CPP and obesity lowest). Independent y-axes are used per panel.

These profiles remained consistent across pre-specified sensitivity analyses. The phenotype contrasts were stable across age (Figure 6) and across alternative age windows spanning 2–14 years, during which the CPP deficits in 25(OH)D (adjusted *d*, −0.26 to −0.28) and vitamin E (*d*, −0.23 to −0.28), as well as the increased odds of vitamin D deficiency (OR 1.36–1.51), were essentially unchanged (Supplementary Table S8). In sex-stratified analysis (Supplementary Figure S1), the obesity–high vitamin A and short-stature–high 25(OH)D signals were evident in both sexes, while the CPP reductions in 25(OH)D and vitamin E were clear in girls but underpowered in boys, among whom CPP was rare (*n* = 84). A broadly defined precocious-puberty analysis that included boys was concordant (Supplementary Table S3). The between-group ordering of 25(OH)D was preserved within every season (Supplementary Figure S2 and Supplementary Table S4). Finally, the higher 25(OH)D in short stature was accompanied by about twice the rate of the vitamin D2 (ergocalciferol) supplementation marker (25(OH)D2: D3 > 0.2) compared with controls (12.1% vs. 6.1%; χ^2^ *p* < 0.0001; Supplementary Figure S3 and Supplementary Table S13; underlying 25(OH)D2, 25(OH)D3 and D2: D3 distributions by group in Supplementary Table S2), and the higher 25(OH)D persisted after excluding girls positive for this marker (Supplementary Table S13). This was unchanged across alternative definitions of the marker: with the ratio set at 0.10, 0.15, 0.20 or 0.30, the adjusted d for short stature in marker-negative girls ranged from +0.24 to +0.25 and the deficiency odds ratio from 0.57 to 0.64, while the CPP estimates ranged from d = −0.26 to −0.28 and OR 1.38 to 1.45 (Supplementary Table S15).

### Unsupervised clustering of fat-soluble vitamins

Two exploratory analyses examined the joint structure of the fat-soluble vitamins. The measured analytes were only weakly correlated (all |*r*| ≤ 0.23; 25(OH)D3 and vitamin E showed the strongest association, *r* = 0.23, and 25(OH)D2 and 25(OH)D3 were weakly inversely related, *r* = −0.11), indicating that the vitamins varied largely independently. Unsupervised K-means clustering of the four measured analytes yielded three clusters (Supplementary Figure S4 and Supplementary Tables S9–S11). Two reflected a single overall concentration axis (uniformly lower versus uniformly higher vitamin levels), while the third (cluster C3; *n* = 63) was qualitatively distinct, defined by markedly elevated 25(OH)D2 together with lower 25(OH)D3, a pattern consistent with exogenous ergocalciferol intake. Cluster membership differed across phenotype groups [χ^2^(6) = 143.1, *p* < 0.0001]: the ergocalciferol cluster was concentrated in short stature (3.7%, roughly three times the 1.2% of healthy controls) and rare in CPP (0.7%) and obesity (0.2%), whereas CPP was overrepresented in the low-vitamin cluster. Because the overall cluster separation was weak (mean silhouette ≈0.25), these clusters are interpreted as descriptive patterns rather than discrete metabolic subtypes.

## Discussion

Our study demonstrated that, within a single large pediatric population, three common pediatric endocrine disorders each carry a distinct fat-soluble vitamin profile rather than a shared deficiency. Relative to healthy controls, girls with central precocious puberty (CPP) had lower 25-hydroxyvitamin D [25(OH)D] (adjusted *d* = −0.26) and lower vitamin E (*d* = −0.25); girls with obesity had markedly higher vitamin A (*d* = +0.50); and girls with short stature had higher 25(OH)D (*d* = +0.26). The vitamin D findings diverged in direction between phenotypes: the odds of vitamin D deficiency were increased in CPP (OR 1.43) but decreased in short stature (OR 0.60), so that the two disorders fell on opposite sides of the healthy reference; the short-stature arm of this divergence may substantially reflect differential supplementation. The main significance of these findings is that the vitamin–phenotype associations are not produced by a single nonspecific feature of being a referred or unwell child, and that their magnitude is modest, in contrast to the large effects implied by earlier, smaller studies. What we observed is not a coordinated shift across all three vitamins but, for each phenotype, one robust direction-specific deviation (higher vitamin A in obesity, lower 25(OH)D and lower vitamin E in CPP, and higher 25(OH)D in short stature). We therefore use “profile” rather than “signature” throughout. The three vitamins jointly added little discrimination beyond age (incremental ΔAUC 0.03–0.05 over age alone; Supplementary Table S12) and the unsupervised clustering was weakly separated (mean silhouette ≈0.25), indicating modest shifts in central tendency with substantial overlap between groups. These patterns should therefore not be interpreted as a multivariate biomarker panel or as clinically discriminating profiles: they do not support the use of a fat-soluble vitamin profile to classify or triage an individual child, and the phrase “phenotype-specific profile” should be read throughout as a statement about group-level distributions rather than about individual-level discrimination. The two borderline associations (CPP–vitamin A and obesity–vitamin E) were not robust on internal validation and should be regarded as preliminary pending external replication.

The direction of our vitamin D finding in CPP is concordant with most previous case–control studies and meta-analyses. Lee et al. (43) reported lower mean 25(OH)D in girls with CPP (17.1 vs. 21.2 ng/mL) and an odds ratio for deficiency of 3.05, and an updated meta-analysis by Cheng et al. (8) found a pooled standardized mean difference of −1.114 in girls and an odds ratio of vitamin D deficiency of 1.531. However, our age-adjusted effect (*d* = −0.26; deficiency OR 1.43) is considerably smaller than the pooled meta-analytic difference of roughly −1.1. This divergence is expected rather than contradictory: the same meta-analysis explicitly reported high heterogeneity and evidence of publication bias (8), small case–control studies are prone to overestimation, and at least one carefully conducted study found no association between vitamin D status and CPP at all (44). Our large sample, analyzed with a single standardized assay, therefore provides a more conservative, less bias-prone estimate of the true association, which we regard as one of the study’s main contributions.

Our spline analysis adds further support: restricted cubic splines showed a nonlinear inverse association between continuous 25(OH)D and CPP with an inflection near 25–30 ng/mL. This closely matches Zhao et al. (45), who, also in a Wuhan cohort, described a nonlinear relationship and identified a serum 25(OH)D cut-off of 31.8 ng/mL for idiopathic CPP. The convergence of two independent samples near the conventional sufficiency boundary is consistent with an association that changes most steeply around this point. The nonlinearity was primarily step-shaped: in internal cross-validation, polynomial expansion of the 25(OH)D term did not improve out-of-sample predictive accuracy, consistent with a pattern in which the odds of CPP are lower above the deficiency boundary and similar across higher concentrations, rather than a graded association across the entire 25(OH)D range.

The most apparent discrepancy with prior work concerns short stature. Whereas Xu et al. (22) reported lower 25(OH)D3 and free 25(OH)D in short-stature children, we observed higher total 25(OH)D and a lower prevalence of deficiency. This difference is partly reconciled by the same study’s central methodological observation: total 25(OH)D [25(OH)D2 + 25(OH)D3] can overestimate vitamin D storage when 25(OH)D2 (ergocalciferol) is present (22). In our short-stature group the marker of D2 supplementation (a 25(OH)D2:25(OH)D3 ratio > 0.2) was about twice as frequent as in controls (12.1% vs. 6.1%), indicating that ergocalciferol supplementation, common among children undergoing growth evaluation and treatment, contributes to their higher total 25(OH)D. The higher 25(OH)D persisted after excluding girls positive for this marker and was also evident for 25(OH)D3 (Supplementary Table S13). This residual difference cannot, however, be read as endogenous. Because cutaneous synthesis and oral cholecalciferol both yield D3, the 25(OH)D2:25(OH)D3 ratio is blind to cholecalciferol (D3) supplementation, and cholecalciferol is the predominant supplemental form prescribed in our setting. Differential supplementation therefore remains a plausible and potentially important contributor to the higher 25(OH)D observed in short stature, although the available data cannot distinguish supplementation effects from phenotype-related or other unmeasured influences. This does not, however, restore a generic “low vitamin D in all referred children” account of the CPP finding: because the short-stature and CPP groups diverge in opposite directions from controls, the CPP vitamin D signal cannot be dismissed as such. Indeed, a nonspecific referral effect need not act in a single direction: clinically referred or chronically ill children have been reported to show either lower 25(OH)D (through indication-related bias) or higher 25(OH)D (because those under regular pediatric follow-up are more likely to be supplemented) (46, 47); thus, such an effect could shift 25(OH)D either way and cannot produce the opposite-direction divergence of CPP and short stature. What that divergence establishes is that the two groups are not displaced by one common referral bias; it does not, on its own, establish that either displacement is biological in origin.

The obesity profile is biologically coherent with established literature. Higher serum vitamin A in obesity is consistent with the well-documented elevation of retinol-binding protein 4 (RBP4), the circulating transport protein for retinol, in childhood obesity; for example, obese children have higher RBP4 than lean children (30.5 vs. 26.3 mg/L), and RBP4 is primarily a marker of adipose-tissue mass (19). The accompanying lower 25(OH)D in obesity is also concordant with the recognized volumetric dilution and adipose sequestration of vitamin D (48, 49). Finally, the lower vitamin E we observed in CPP is, to our knowledge, the first systematic report on the precocious side of the growth-disorder spectrum. An altered oxidant–antioxidant balance has been described in girls with CPP (15), and as the principal lipid-soluble antioxidant, vitamin E is plausibly involved; however, lower circulating vitamin E could equally reflect dietary, lipid-related, or consumption differences, and our cross-sectional data cannot establish its basis. In particular, because circulating *α*-tocopherol is strongly lipid-dependent and is conventionally interpreted relative to serum lipids (for example, the tocopherol-to-cholesterol ratio), and we measured total α-tocopherol without concurrent lipid profiles, the lower vitamin E in CPP may partly reflect differences in circulating lipids rather than vitamin E status per se and should be regarded as hypothesis-generating pending lipid-adjusted analysis.

The pathways that might connect fat-soluble vitamin status to these phenotypes are not tested by our design, but they are specific enough to be stated explicitly, and we set them out here as hypotheses rather than as inferences from our data. For vitamin D and pubertal onset, the proximate event initiating puberty is reactivation of pulsatile gonadotropin-releasing hormone (GnRH) secretion, driven by kisspeptin-expressing KNDy neurons of the arcuate nucleus signaling through KISS1R/GPR54 (50, 51). Several nodes of this circuit are, in principle, vitamin D-sensitive. VDR and 1α-hydroxylase are expressed in the human hypothalamus (6), so 1,25-dihydroxyvitamin D could act locally as a transcriptional modulator of neuroendocrine gene expression; kisspeptin signaling is Gq/11-coupled and calcium-dependent, and vitamin D is the principal regulator of extracellular calcium homeostasis (1), providing a second, calcium-dependent route by which vitamin D status could influence GnRH pulse generation. Downstream, VDR ablation reduces gonadal aromatase (CYP19A1) expression and activity with impaired folliculogenesis and hypergonadotropic hypogonadism (52), implicating vitamin D in steroid-hormone biosynthesis, and VDR-null animals also show post-weaning growth retardation (53), situating vitamin D at the growth–reproduction interface relevant to both phenotypes examined here. Finally, vitamin D is a broad regulator of innate and adaptive immunity and of inflammatory transcription (4), and low-grade inflammation has been proposed as a permissive signal for early pubertal activation. A prospective association between vitamin D deficiency and earlier menarche (54) is directionally consistent with our cross-sectional finding. None of these mechanisms can be evaluated without hypothalamic, gonadal, or inflammatory measurements, and our data are compatible with, but do not support, any of them individually.

For obesity, volumetric dilution and adipose sequestration are commonly emphasized (48, 49), but these are probably not the only, and possibly not the principal, mechanisms. Hepatic CYP2R1, the main vitamin D 25-hydroxylase, is down-regulated in diet-induced obesity, and obese animals generate less 25(OH)D per unit of substrate (55), so reduced activation capacity operates alongside altered distribution. Obese adipose tissue is also a site of chronic low-grade inflammation with macrophage infiltration and adipokine dysregulation: 1,25-dihydroxyvitamin D abolishes macrophage-induced NF-κB and MAPK activation and chemokine release in human adipocytes (56), placing vitamin D within rather than merely downstream of the obesity–inflammation axis, while insulin resistance is itself promoted by the adipocyte-derived retinol carrier RBP4 (57), which connects the vitamin A and vitamin D findings in this group to a shared adipose–endocrine mechanism. Direction of effect is relevant here: bi-directional Mendelian randomization indicates that higher body-mass index lowers 25(OH)D, whereas genetically lower 25(OH)D has little effect on adiposity (58). This supports interpreting our obesity–vitamin D association as adiposity acting on vitamin D status rather than the converse, and reinforces the reverse-causation caveat that we raise for CPP.

The lower vitamin E in girls with CPP invites an oxidative-stress interpretation, which we set out with its limits. Puberty is accompanied by a measurable shift in oxidant–antioxidant balance (15, 16), and *α*-tocopherol is the principal chain-breaking antioxidant of membranes and lipoproteins, terminating lipid peroxidation and limiting propagation of radical damage to polyunsaturated fatty acids and mitochondrial membranes (3). *α*-Tocopherol additionally has non-antioxidant regulatory functions – inhibition of protein kinase C and modulation of NF-κB- and AP-1-dependent transcription and antioxidant gene expression (7) – so a lower circulating concentration could mark either increased consumption in a pro-oxidant state or altered redox-sensitive signaling. A shared inflammatory–redox axis is plausible, since 1,25-dihydroxyvitamin D also restrains NF-κB activation (56), which would offer one route by which the vitamin D and vitamin E deficits we observed in the same phenotype could be related rather than independent. Two caveats prevent us from adopting this reading. First, no marker of oxidative stress (for example malondialdehyde, F2-isoprostanes, or total antioxidant capacity) was measured in this cohort, so the redox interpretation has no direct support in our data. Second, circulating α-tocopherol is lipoprotein-transported and is conventionally interpreted relative to serum lipids (59), and we lacked concurrent lipid profiles; lipid-standardized replication is therefore a prerequisite before any redox mechanism is invoked.

For vitamin A, the obesity finding has so far been related to RBP4 alone; the wider molecular context is that retinol is the precursor of retinoic acid, which signals through RAR–RXR heterodimers to control cellular differentiation, adipogenesis and metabolic gene programs (5, 60). Because RXR is also the obligate partner of the VDR, retinoid and vitamin D signaling compete for and converge on shared nuclear-receptor machinery (5), which is a molecular rationale for measuring these vitamins together rather than separately, even though our data cannot demonstrate such crosstalk. In obesity specifically, RBP4 is secreted by adipocytes in proportion to fat mass and acts as an insulin-resistance-promoting adipokine (19, 57), and its concentrations track both adiposity and pubertal stage in children (19, 21). The higher serum retinol we observed in obesity is therefore most plausibly a transport-related phenomenon reflecting elevated RBP4 rather than an expanded vitamin A store, since circulating retinol is homeostatically regulated and is a poor index of hepatic reserves (60) – an interpretation that also explains why vitamin A deficiency was least frequent in the obesity group despite no evidence of higher intake. Testing this, and the possibility that retinoic acid signaling contributes to the adipose phenotype, would require measurement of RBP4, retinol-binding stoichiometry and retinoic acid-responsive transcripts, none of which was available to us.

The principal strength of this study is its scale and analytical uniformity: a large single-center sample in which vitamins A, D, and E were measured simultaneously by a single standardized HPLC assay, eliminating the inter-assay and inter-method variability that drives heterogeneity in the meta-analytic literature. The multi-phenotype design embeds a built-in opposite-direction control (short stature), allowing a single common referral bias to be excluded directly rather than assumed. We adjusted for age throughout, controlled the false-discovery rate across the many comparisons, modeled the shape of the 25(OH)D–phenotype association with restricted cubic splines, and conducted extensive pre-specified sensitivity analyses across sex, season, age window, and obesity stratum. The 25(OH)D2:25(OH)D3 ratio further allowed us to probe supplementation without dedicated records. An unsupervised clustering of the measured analytes independently recovered this ergocalciferol-supplementation subgroup, concentrated in short stature (Supplementary Figure S4). Finally, an internal cross-validation program (bootstrap resampling, repeated 10-fold cross-validation, and leave-one-season-out validation) confirmed that the five principal profile effects were directionally stable across all resampling schemes (Supplementary Table S12), whereas the two borderline effects (CPP–vitamin A and obesity–vitamin E) warrant cautious interpretation and external replication. Across phenotypes, age alone already discriminated cases from controls (age-only AUC 0.67 for CPP, 0.54 for short stature, and 0.72 for obesity), and adding the three vitamins improved discrimination only modestly (to 0.70, 0.58, and 0.77; incremental ΔAUC +0.03, +0.04, and +0.05; Supplementary Table S12), indicating that the vitamins contribute limited additional separation beyond age. The modest discriminative accuracy for short stature (AUC 0.58) is expected rather than problematic: this group deviated from controls on a single vitamin (25(OH)D, a difference that may itself substantially reflect supplementation) with no alteration in vitamins A or E, so a three-vitamin classifier necessarily has limited separation, and the low AUC is itself consistent with a narrow, single-analyte difference rather than a broad metabolic shift.

The generalizability of these estimates is bounded in three respects. First, geography: all children were recruited in Wuhan, at approximately 30.5° N, where cutaneous vitamin D synthesis is possible for most of the year; the absolute 25(OH)D distribution, and therefore the position of the 25–30 ng/mL inflection, would be expected to shift at higher latitudes or in populations with markedly different sun-exposure behavior. Second, ethnicity: the cohort is predominantly Han Chinese, and both skin pigmentation and dietary patterns differ across ethnic groups in ways that affect 25(OH)D and retinol concentrations. Third, sex: the primary analysis was restricted to girls, and although the obesity and short-stature signals were evident in both sexes on stratified analysis (Supplementary Figure S1), the CPP estimates in boys were underpowered and no inference about boys should be drawn from them. The inflection point near 25–30 ng/mL should therefore be read as a feature of this population rather than as a transferable threshold, and external replication at other latitudes, in other ethnic groups and in boys is required before any of these values is generalized.

Several limitations must be acknowledged. First, the cross-sectional design precludes causal inference and cannot exclude reverse causation, since advancing puberty or adiposity could lower 25(OH)D rather than the converse. Second, this was a single-center dataset from an urban tertiary children’s hospital; although the control group comprised children attending for a routine health examination rather than for a clinical indication related to the study phenotypes, both cases and controls were necessarily drawn from children who underwent fat-soluble vitamin testing during their visit, and this tested subset may differ from the general pediatric population (for example, in family health-consciousness or care-seeking), so the controls are not population-representative and residual selection effects cannot be excluded. Quantitatively, 57,433 of the 68,424 eligible children (84%) met no study-group definition and were not analyzed, so the reference distribution reported here characterizes tested children at a tertiary center rather than the source pediatric population, and every between-group estimate should be read as relative to this tested subset. Although the opposite-direction divergence of CPP and short stature argues against a uniform bias affecting all referred children, it does not exclude the possibility that the control group itself, tested for indications that were not recorded, provides a shifted reference, so all between-group estimates relative to controls should be interpreted with this caveat. Specifically, families who present a well child for a routine health examination that includes fat-soluble vitamin testing may differ from the general population in socioeconomic position, dietary quality and use of vitamin supplements, none of which was recorded. If such families were more likely to use vitamin supplements, the resulting bias would be toward higher 25(OH)D in controls and would tend to exaggerate the CPP deficit and attenuate the short-stature elevation; such a bias cannot, however, generate the opposite-direction divergence of CPP and short stature, since both are compared with the same reference group. Third, supplementation was inferred from the D2: D3 marker rather than documented intake, and we lacked dietary and supplement records. We also had no data on dietary intake of lipid-rich or fortified foods, on physical activity, or on daily sunlight exposure, all of which are determinants of fat-soluble vitamin status and none of which is likely to be balanced across the phenotype groups: children under regular endocrine follow-up may have systematically different diets, greater indoor time and different supplement use from healthy children attending for a single examination, so residual confounding by these unmeasured behavioral exposures cannot be excluded and may act in either direction. This is most consequential for the short-stature 25(OH)D elevation, which we already interpret as plausibly reflecting differential supplementation, and least consequential for the CPP-versus-short-stature divergence, which would require these exposures to differ in opposite directions between two clinically followed groups. Fourth, the dataset did not include Tanner staging, bone age or pubertal hormone concentrations, measured height and weight were recoverable only in part (see below), and phenotypes were defined from clinical diagnoses rather than from standardized re-ascertainment, so some diagnostic misclassification is possible (for example, central precocious puberty was based on the recorded clinical diagnosis, and confirmation by gonadotropin-releasing hormone [GnRH] stimulation testing could not be systematically verified, so some cases may represent premature thelarche or peripheral precocity, which would bias effect estimates toward the null). To the extent that girls with premature thelarche or peripheral precocity misassigned to the CPP group more closely resemble controls than true CPP cases, any such misclassification would tend to attenuate the contrast and bias the CPP estimates toward the null, in which case the CPP associations reported here would be conservative. The short-stature group is also etiologically heterogeneous, comprising growth hormone deficiency, idiopathic short stature, small-for-gestational-age status and other causes, which could not be separated reliably from the recorded diagnoses; this matters for the vitamin D finding in particular, because children under growth hormone treatment may be more likely to receive calcium and vitamin D supplementation, so aggregating etiologies may both mask cause-specific associations and average a supplementation-related elevation across treated and untreated children. Tanner staging was likewise unavailable, so pubertal stage among the older control girls could not be adjusted for directly; because serum retinol and its transport protein rise with advancing pubertal stage, unmeasured variation in maturation within the 8–13-year control stratum could contribute to residual confounding, particularly for vitamin A. As a partial check, the primary contrasts were repeated separately in girls aged 3 to under 8 years, who are prepubertal by age, and in girls aged 8–13 years; the direction and approximate magnitude of every principal effect were preserved in both strata (Supplementary Table S8B), although chronological age remains an imperfect surrogate for pubertal stage. For adiposity, measured height and weight were retrieved for this revision and used to adjust the primary contrasts for a continuous covariate (Supplementary Table S16); weight was recoverable for 86.3% of the cohort, and analyzable after a plausibility screen for 86.1%, but height for only 24.1%, so BMI-based adjustment rests on a subgroup that is underpowered, and weight-based adjustment is imperfect in the short-stature group because weight-for-age partly encodes the phenotype itself. Residual confounding by adiposity is therefore reduced but not eliminated. Fifth, the primary analysis was restricted to girls because CPP is rare in boys, in whom the relevant estimates were underpowered. Sixth, age (the principal confounder, given the marked between-group age differences) was modeled as a linear covariate in the primary effect-size models, and because the vitamin–age relationships are not strictly linear (Figure 6), residual confounding by age cannot be fully excluded. The phenotype contrasts were nonetheless stable across alternative age windows (Supplementary Table S8), and a sensitivity analysis modeling age with restricted cubic splines (Supplementary Table S14) confirmed that the five principal direction-specific effects retained their direction and statistical significance; the borderline CPP–vitamin A association, however, strengthened under spline adjustment, so this residual age effect is most relevant for the vitamin A finding. Seventh, our HPLC assay did not separately quantify the C3-epimer of 25(OH)D (3-epi-25(OH)D3), which can co-elute with and inflate measured total 25(OH)D, particularly in younger children; this could lead to a modest overestimation of 25(OH)D in the lower age range, although it would be expected to affect all phenotype groups similarly and therefore is unlikely to explain the between-group contrasts. Finally, the effect sizes were small to moderate, so the clinical relevance of any single vitamin measurement in an individual child is limited.

Clinically, these findings caution against over-interpreting an isolated low 25(OH)D value as a causal factor in a child with CPP: the association is real but modest and phenotype-specific, and vitamin D results should be read in the context of possible supplementation and of the specific disorder. The inflection near 30 ng/mL is consistent with conventional sufficiency targets and offers no argument for a different cut-off in this setting.

Beyond these immediate cautions, the translational significance of phenotype-specific rather than uniform profiles is conditional at this stage. If prospective work confirms that these patterns are stable and at least partly causal, assessment of fat-soluble vitamins in pediatric endocrine practice could become phenotype-directed rather than uniform: 25(OH)D would be interpreted against the child’s diagnosis and documented supplementation rather than against a single population threshold; vitamin E, lipid-standardized, might be evaluated in girls under investigation for precocious puberty; and serum retinol in obesity would be read as a marker of adipose RBP4 rather than of vitamin A sufficiency. Expressed in the language of precision nutrition and nutrigenomics, the longer-term goal would be to determine which children benefit from correction of a given micronutrient on the basis of phenotype, genotype (for example VDR or CYP2R1 variants) and biomarker profile, rather than on an isolated laboratory value (61, 62). The present data do not license any of this. With an incremental ΔAUC of 0.03–0.05, weak unsupervised separation, and an unmeasured supplementation contribution to the largest vitamin D contrast, our findings describe population-level patterns and do not constitute a biomarker-guided decision rule; we specifically do not recommend routine vitamin A or vitamin E screening in these disorders, nor vitamin D supplementation aimed at modifying pubertal timing, on the basis of this study.

These limits define the research agenda. First, longitudinal cohorts with serial vitamin measurement, Tanner staging, bone age, gonadotropin data and documented supplementation are needed to establish whether the vitamin differences precede or follow the phenotype. Second, Mendelian randomization using established genetic instruments for 25(OH)D and for pubertal timing – several hundred independent loci for age at menarche are now available (63) – could address causality without the confounding and reverse causation inherent in cross-sectional designs, and intervention trials remain the only way to determine whether correcting deficiency alters pubertal progression or growth. Third, mechanistic experiments (hypothalamic and gonadal VDR manipulation with kisspeptin–GnRH readouts, adipocyte–macrophage co-culture, and lipid-standardized tocopherol with direct oxidative-stress markers) would test the specific pathways outlined above. Fourth, integrated multi-omics – genomic and epigenomic profiling of vitamin-responsive loci, transcriptomics and proteomics of adipose and immune compartments, and targeted metabolomics and lipidomics – would allow the modest serum differences reported here to be connected to the molecular programs that might generate them (61). Replication in boys, in other ethnic and geographic populations, and with free 25(OH)D and separate D2/D3 and C3-epimer quantification, remains a prerequisite throughout.

## Conclusion

In girls, common pediatric endocrine disorders are accompanied by distinct, phenotype-specific fat-soluble vitamin profiles rather than a uniform deficiency of the unwell child: CPP and short stature fall on opposite sides of the healthy reference for vitamin D, a pattern that no single, uniformly acting referral bias could produce. The short-stature elevation, however, may substantially reflect differential vitamin D supplementation (including cholecalciferol, which our supplementation marker cannot detect), and the modest magnitude of all effects tempers the strong causal interpretations of earlier, smaller reports. Fat-soluble vitamin status in these conditions may therefore be better regarded as a phenotype-associated biomarker than an established causal factor: one to be interpreted cautiously, in the light of supplementation, and pursued through prospective and mechanistic research. Because the individual effects are small and add little discrimination beyond age, these findings should be read as phenotype-associated population-level patterns rather than as clinically discriminating profiles, and the molecular pathways we outline remain hypotheses to be tested prospectively and experimentally.

## Data Availability

The original contributions presented in the study are included in the article/Supplementary material, further inquiries can be directed to the corresponding author/s.
