# Supplementary material for: Phenotype-specific 25-hydroxyvitamin D and fat-soluble vitamin profiles in girls with pediatric endocrine disorders: a large cross-sectional study
Source: Front Nutr. 2026 Aug 28;13:1924137. doi: 10.3389/fnut.2026.1924137 (PMC13562236; doi:10.3389/fnut.2026.1924137)
Supplement: Supplementary file 1 [file Supplementary_file_1.DOCX]

**Supplementary Figure S1. Fat-soluble vitamin profiles by sex (sensitivity analysis).** Pre-specified sensitivity analysis including both sexes (the primary analysis was restricted to girls; see Methods). Age-adjusted standardized mean differences (Cohen’s *d*) in serum vitamin A, total 25(OH)D, and vitamin E for each phenotype group relative to same-sex healthy controls, for boys and girls aged 3–13 years with complete measurements. Boys: healthy control *n* = 2,298; CPP *n* = 84; short stature *n* = 1,418; obesity *n* = 1,049. Girls: healthy control *n* = 1,503; CPP *n* = 1,146; short stature *n* = 970; obesity *n* = 492. Estimation, color scale, and significance markers are as in Figure 2 (age-adjusted group coefficient; reference *d* = 0; symmetric scale −0.55 to +0.55; asterisks denote Benjamini–Hochberg false-discovery-rate–adjusted *P* within each sex panel: **P* < 0.05, ***P* < 0.01, ****P* < 0.001, *****P* < 0.0001; ns, not significant). The girls panel reproduces the primary analysis (Figure 2). The obesity–high vitamin A signal (boys +0.42, girls +0.50) and the short-stature–high 25(OH)D signal (boys +0.17, girls +0.26) are evident in both sexes. The CPP reductions in 25(OH)D and vitamin E are clear in girls but do not reach significance in boys, in whom CPP is rare (*n* = 84) and estimates are correspondingly underpowered—this imprecision is the rationale for restricting the primary analysis to girls.

**Supplementary Figure S2. Seasonal variation in serum 25(OH)D by phenotype group.** Boxplots (median, interquartile range, and 1.5×IQR whiskers; points beyond are outliers) of total serum 25-hydroxyvitamin D [25(OH)D] by meteorological season of blood sampling, within each phenotype group, among girls aged 3–13 years in the primary cohort (healthy control, *n* = 1,503; CPP, *n* = 1,146; short stature, *n* = 970; obesity, *n* = 492; Wuhan, Northern Hemisphere). The *y*-axis is truncated at 80 ng/mL for clarity (two values up to 152 ng/mL are not shown); box and whisker statistics are computed from all observations. 25(OH)D is modestly higher in summer and autumn than in spring and winter, consistent with seasonal sunlight exposure. The between-group ordering is stable across all seasons—short stature highest, CPP and obesity lowest, healthy controls intermediate—indicating that the phenotype differences in 25(OH)D are not driven by season; season was nonetheless included as a covariate in sensitivity model M3, and the formal season-stratified analysis of vitamin D deficiency is reported in Table S4.

**Supplementary Figure S3. Vitamin D_2_ (ergocalciferol) supplementation marker by phenotype group.** Proportion of children with a serum 25(OH)D_2_-to-25(OH)D_3_ ratio greater than 0.2—a marker of ergocalciferol (vitamin D_2_) supplementation, since endogenously synthesized vitamin D is exclusively D_3_—by phenotype group, among girls aged 3–13 years with both D_2_ and D_3_ measured (healthy control, *n* = 1,503; CPP, *n* = 1,146; short stature, *n* = 970; obesity, *n* = 492). The proportion differed across groups (χ² *P* < 0.0001) and was about twice as high in short stature (12.1%) as in healthy controls (6.1%; short stature vs control, χ² *P* < 0.0001), whereas CPP (5.8%) and obesity (3.9%) were similar to or lower than controls. The elevated D_2_-supplementation rate in short stature is consistent with supplementation contributing to their higher total 25(OH)D and lower odds of vitamin D deficiency (Figures 2 and 6)—that is, the short-stature “high vitamin D” signal likely reflects treatment rather than the phenotype itself; accordingly, the opposite displacement of the two groups should be read only as excluding a single common referral bias, and does not in itself establish that either displacement is biological in origin.

**Supplementary Figure S4. Unsupervised clustering of serum fat-soluble vitamin profiles by phenotype group.** Unsupervised K-means clustering of standardized serum concentrations of the four directly measured fat-soluble vitamin analytes—vitamin A, 25(OH)D_2_, 25(OH)D_3_, and vitamin E—among girls aged 3–13 years in the primary cohort (healthy control, *n* = 1,503; CPP, *n* = 1,146; short stature, *n* = 970; obesity, *n* = 492). Total 25(OH)D was deliberately excluded from clustering because it is the arithmetic sum of 25(OH)D_2_ and 25(OH)D_3_ and would introduce exact collinearity; it is shown only as a derived quantity in Table S9. The number of clusters (*k* = 3) was selected by convergent elbow and silhouette criteria (Table S11); the overall silhouette width was low (≈0.25), indicating weak global cluster separation, so the analysis is reported as a hypothesis-free corroboration of the supplementation pattern rather than as evidence of discrete metabolic phenotypes. Clusters are ordered and labeled (C1–C3) by ascending mean total 25(OH)D. (A) Cluster profiles, shown as the mean standardized concentration (*z*-score) of each clustered analyte within each cluster. C1 shows uniformly lower vitamin concentrations and C2 uniformly higher concentrations (a single overall-level axis), whereas C3 is qualitatively distinct: it is defined by markedly elevated 25(OH)D_2_ (mean *z* ≈ +6) together with lower 25(OH)D_3_ (*z* ≈ −0.8)—the hallmark of exogenous ergocalciferol (D_2_) intake rather than endogenous synthesis. The color scale is capped at ±1 (out-of-range values squished to the limit); the 25(OH)D_2_ value in C3 exceeds the scale and is displayed at the maximum, with its true value printed in the cell. (B) Within-phenotype cluster composition, showing the proportion of each phenotype group assigned to each cluster. The distribution of clusters differed significantly across phenotype groups (χ²(6) = 143.1, *P* < 0.0001). The D_2_-supplementation cluster (C3) was concentrated in short stature (3.7%), roughly three times the rate in healthy controls (1.2%), and rare in CPP (0.7%) and obesity (0.2%), whereas CPP was over-represented in the low-vitamin cluster C1. That an unsupervised, label-free, threshold-free method spontaneously isolated an ergocalciferol-supplementation cluster enriched in short stature corroborates the interpretation that the higher total 25(OH)D of the short-stature group reflects D_2_ supplementation rather than the phenotype itself (cf. Supplementary Figure S3); accordingly, the opposite displacement of the two groups should be read only as excluding a single common referral bias, and does not in itself establish that either displacement is biological in origin.
