# Supplementary material for: Phenotype-specific 25-hydroxyvitamin D and fat-soluble vitamin profiles in girls with pediatric endocrine disorders: a large cross-sectional study
Source: Front Nutr. 2026 Aug 28;13:1924137. doi: 10.3389/fnut.2026.1924137 (PMC13562236; doi:10.3389/fnut.2026.1924137)
Supplement: Supplementary file 2 [file Supplementary_file_2.DOCX]

**Supplementary Tables**

*Every table listed below is cited at least once in the main text, and every supplementary table cited in the main text appears below; the third column gives the section(s) of the revised manuscript in which each table is cited. Tables S15 and S16, and Panel B of Table S8, are new in this revision.*

| **Table** | **Title** | **Cited in** |
| --- | --- | --- |
| Table S1 | Sequential adjustment of vitamin D deficiency odds for CPP and short stature | Results |
| Table S2 | 25(OH)D_2_, 25(OH)D_3_, and D_2_:D_3_ ratio by phenotype group | Results |
| Table S3 | Sensitivity: broadly-defined precocious puberty (including boys) and vitamin D deficiency | Results |
| Table S4 | Vitamin D deficiency prevalence by group, sex, and season | Results |
| Table S5 | Vitamin D deficiency odds in CPP stratified by obesity status | Results |
| Table S6 | Direct contrast of CPP versus short stature, age-adjusted | Results, Figure legends |
| Table S7 | Full pairwise between-group comparison matrix | Results, Figure legends |
| Table S8 | Robustness of the CPP profile to the choice of age window | Results, Discussion |
| Table S9 | Serum fat-soluble vitamin profiles of the K-means clusters | Results |
| Table S10 | Distribution of K-means clusters across phenotype groups | Results |
| Table S11 | Selection of the number of clusters | Results |
| Table S12 | Internal cross-validation of the primary fat-soluble vitamin profiles | Methods, Results, Discussion |
| Table S13 | Sensitivity of the short-stature 25(OH)D finding to D_2_ (ergocalciferol) supplementation | Results, Discussion |
| Table S14 | Sensitivity of primary effect sizes to non-linear (spline) age adjustment | Discussion |
| Table S15 | Sensitivity of the D_2_-supplementation analysis to the choice of 25(OH)D_2_:D_3_ threshold | Methods, Results |
| Table S16 | Sensitivity of the primary phenotype contrasts to continuous adjustment for measured adiposity | Methods, Results, Discussion |

**Table S1. Sequential adjustment of vitamin D deficiency odds for CPP and short stature.**

| **Model** | **CPP - OR (95% CI), p** | **Short stature - OR (95% CI), p** |
| --- | --- | --- |
| M1: crude | 1.95 (1.65-2.31), <0.001 | 0.68 (0.56-0.83), <0.001 |
| M2: + age | 1.42 (1.19-1.70), <0.001 | 0.60 (0.48-0.74), <0.001 |
| M3: + age + season + obesity | 1.33 (1.10-1.61), 0.004 | 0.53 (0.42-0.66), <0.001 |

*Logistic regression for vitamin D deficiency (25(OH)D <=20 ng/mL); control as reference. Obesity group excluded so obesity could be modeled as a covariate; the M2 (age-adjusted) CPP estimate of 1.42 therefore differs trivially from the primary age-adjusted estimate of 1.43 (Table 2), which is fitted in the full four-group cohort. Profile-likelihood CIs.*

**Table S2. 25(OH)D_2_, 25(OH)D_3_, and D_2_:D_3_ ratio by phenotype group.**

| **Group** | **25(OH)D_2_** | **25(OH)D_3_** | **D_2_:D_3_ ratio** |
| --- | --- | --- | --- |
| Control | 0.95 (0.48-1.66) | 25.02 (18.62-31.71) | 0.04 (0.02-0.07) |
| CPP | 0.97 (0.51-1.63) | 20.74 (15.42-25.34) | 0.05 (0.02-0.09) |
| Short stature | 1.22 (0.64-2.19) | 25.51 (19.50-31.85) | 0.05 (0.02-0.10) |
| Obesity | 0.97 (0.53-1.52) | 20.14 (16.06-25.40) | 0.05 (0.03-0.09) |

*Median (IQR), ng/mL (ratio dimensionless). Kruskal-Wallis P: 25(OH)D_2_ <0.001; 25(OH)D_3_ <0.001; D_2_:D_3_ <0.001.*

**Table S3. Sensitivity: broadly-defined precocious puberty (including boys) and vitamin D deficiency.**

| **Comparison** | **OR (95% CI)** | **P value** |
| --- | --- | --- |
| Precocious puberty (any, incl. boys) vs control | 1.47 (1.28-1.68) | <0.001 |

*Odds ratio for vitamin D deficiency adjusted for age, sex, and season, vs control. The standardized effect for continuous 25(OH)D was d = -0.25 (precocious-any vs control), consistent with the girls-only analysis.*

**Table S4. Vitamin D deficiency prevalence by group, sex, and season.**

| **Group** | **Sex** | **Spring** | **Summer** | **Autumn** | **Winter** |
| --- | --- | --- | --- | --- | --- |
| Control | F | 36.0 (375) | 15.5 (580) | 20.1 (278) | 32.6 (270) |
| Control | M | 23.2 (577) | 9.3 (905) | 11.2 (394) | 30.8 (422) |
| CPP | F | 50.0 (282) | 20.3 (316) | 28.3 (322) | 65.9 (226) |
| CPP | M | 36.4 (22) | 19.2 (26) | 62.5 (8) | 64.3 (28) |
| Short stature | F | 25.8 (256) | 8.6 (337) | 9.9 (191) | 33.3 (186) |
| Short stature | M | 16.3 (392) | 7.1 (518) | 7.6 (225) | 31.8 (283) |
| Obesity | F | 63.0 (119) | 19.8 (172) | 31.4 (102) | 56.6 (99) |
| Obesity | M | 45.1 (244) | 15.4 (383) | 21.7 (207) | 57.7 (215) |

*Each cell: percentage with vitamin D deficiency (25(OH)D <=20 ng/mL), stratum n in parentheses. Boys' CPP strata are small.*

**Table S5. Vitamin D deficiency odds in CPP stratified by obesity status.**

| **Stratum (vs healthy control)** | **Adjusted OR (95% CI)** | **P value** |
| --- | --- | --- |
| CPP - all (n=1,146) | 1.43 (1.20-1.70) | <0.001 |
| CPP - non-obese (n=1,018) | 1.38 (1.15-1.65) | <0.001 |
| CPP - obese (n=128) | 1.75 (1.20-2.57) | 0.004 |
| Obesity, no CPP (n=492) | 1.23 (0.98-1.55) | 0.076 |

*Age-adjusted ORs for vitamin D deficiency vs the same control group. CPP-all and Obesity rows use the joint all-groups model (= Table 2 / Figure 6, profile-likelihood CI); the two CPP strata are stratum-specific (Wald CI). CPP stays elevated in the non-obese stratum.*

**Table S6. Direct contrast of CPP versus short stature, age-adjusted.**

| **Measure (CPP vs short stature)** | **Estimate (95% CI)** | **P value** |
| --- | --- | --- |
| Vitamin A - adjusted d | +0.01 (-0.08, 0.10) | 0.789 |
| 25(OH)D (total) - adjusted d | -0.54 (-0.61, -0.46) | <0.001 |
| Vitamin E - adjusted d | -0.33 (-0.42, -0.24) | <0.001 |
| Vitamin D deficiency - adjusted OR | 2.40 (1.95-2.97) | <0.001 |

*Short stature is the reference; models adjusted for age. Negative = CPP lower than short stature. The groups diverge most on 25(OH)D.*

**Table S7. Full pairwise between-group comparison matrix.**

| **Vitamin** | **KW omnibus P** | **Comparison** | **Median difference** | **P (BH-adjusted)** |
| --- | --- | --- | --- | --- |
| Vitamin A | 7.3e-23 | Control vs CPP | -5.8 | 2.1e-02 |
| Vitamin A | 7.3e-23 | Control vs Short stature | -2.1 | 3.3e-01 |
| Vitamin A | 7.3e-23 | Control vs Obesity | -37.2 | 7.7e-23 |
| Vitamin A | 7.3e-23 | CPP vs Short stature | 3.7 | 2.3e-01 |
| Vitamin A | 7.3e-23 | CPP vs Obesity | -31.4 | 1.3e-14 |
| Vitamin A | 7.3e-23 | Short stature vs Obesity | -35.1 | 2.3e-18 |
| 25(OH)D (total) | 3.0e-80 | Control vs CPP | 4.4 | 3.5e-40 |
| 25(OH)D (total) | 3.0e-80 | Control vs Short stature | -1.3 | 3.0e-05 |
| 25(OH)D (total) | 3.0e-80 | Control vs Obesity | 5.0 | 1.1e-23 |
| 25(OH)D (total) | 3.0e-80 | CPP vs Short stature | -5.6 | 2.3e-59 |
| 25(OH)D (total) | 3.0e-80 | CPP vs Obesity | 0.6 | 7.8e-01 |
| 25(OH)D (total) | 3.0e-80 | Short stature vs Obesity | 6.3 | 9.0e-38 |
| Vitamin E | 3.2e-25 | Control vs CPP | 841.8 | 1.2e-19 |
| Vitamin E | 3.2e-25 | Control vs Short stature | -28.3 | 6.6e-01 |
| Vitamin E | 3.2e-25 | Control vs Obesity | 655.1 | 2.3e-06 |
| Vitamin E | 3.2e-25 | CPP vs Short stature | -870.1 | 3.2e-18 |
| Vitamin E | 3.2e-25 | CPP vs Obesity | -186.7 | 3.9e-02 |
| Vitamin E | 3.2e-25 | Short stature vs Obesity | 683.4 | 1.8e-06 |

*Omnibus Kruskal-Wallis per vitamin, then pairwise Wilcoxon rank-sum with Benjamini-Hochberg correction. Median difference is first minus second group.*

**Table S8. Robustness of the CPP profile to the choice of age window.**

**Panel A. Alternative age windows.**

| **Age window (y)** | **N** | **CPP / Control n** | **25(OH)D - adj. d (95% CI)** | **Vitamin E - adj. d (95% CI)** | **Vitamin A - adj. d (95% CI)** | **Vit D deficiency - OR (95% CI)** |
| --- | --- | --- | --- | --- | --- | --- |
| 3-13 (primary) | 4,111 | 1,146 / 1,503 | -0.26 (-0.33, -0.20) | -0.25 (-0.33, -0.18) | +0.06 (-0.02, 0.13) | 1.43 (1.20-1.70) |
| 2-14 | 4,484 | 1,148 / 1,793 | -0.28 (-0.34, -0.21) | -0.28 (-0.35, -0.20) | +0.01 (-0.07, 0.08) | 1.47 (1.24-1.75) |
| 4-12 | 3,616 | 1,131 / 1,155 | -0.27 (-0.34, -0.19) | -0.23 (-0.31, -0.14) | +0.12 (0.04, 0.20) | 1.41 (1.18-1.69) |
| 2-12 | 4,314 | 1,136 / 1,715 | -0.26 (-0.32, -0.19) | -0.26 (-0.34, -0.18) | +0.06 (-0.02, 0.14) | 1.51 (1.26-1.81) |
| 4-14 | 3,786 | 1,143 / 1,233 | -0.26 (-0.33, -0.19) | -0.23 (-0.31, -0.15) | +0.09 (0.02, 0.17) | 1.36 (1.14-1.62) |

*Each row re-derives the primary cohort for the stated age window and reports the age-adjusted CPP-vs-control effect using the same models as the main analysis.*

**Table S8, Panel B. Phenotype contrasts estimated separately below and above 8 years of age (age as a proxy for pubertal stage).**

| **Age stratum and group** | **N** | **Group / Control n** | **25(OH)D - adj. d (95% CI)** | **Vitamin E - adj. d (95% CI)** | **Vitamin A - adj. d (95% CI)** | **Vit D deficiency - OR (95% CI)** |
| --- | --- | --- | --- | --- | --- | --- |
| 3 to <8 y – CPP | 2,247 | 502 / 984 | −0.21 (−0.32, −0.10) | −0.21 (−0.33, −0.09) | +0.06 (−0.06, +0.18) | 1.29 (0.96–1.74) |
| 3 to <8 y – Short stature | 2,247 | 610 / 984 | +0.26 (+0.17, +0.35) | +0.01 (−0.09, +0.11) | +0.13 (+0.03, +0.23) | 0.59 (0.42–0.82) |
| 3 to <8 y – Obesity | 2,247 | 151 / 984 | −0.13 (−0.29, +0.03) | +0.08 (−0.10, +0.25) | +0.56 (+0.39, +0.74) | 1.17 (0.76–1.79) |
| 8–13 y – CPP | 1,864 | 644 / 519 | −0.17 (−0.28, −0.05) | −0.16 (−0.28, −0.05) | +0.35 (+0.24, +0.47) | 1.24 (0.97–1.57) |
| 8–13 y – Short stature | 1,864 | 360 / 519 | +0.35 (+0.22, +0.48) | +0.12 (−0.02, +0.25) | −0.06 (−0.19, +0.07) | 0.59 (0.45–0.79) |
| 8–13 y – Obesity | 1,864 | 341 / 519 | −0.11 (−0.25, +0.02) | −0.09 (−0.23, +0.05) | +0.59 (+0.46, +0.72) | 1.14 (0.86–1.50) |

**Table S9. Serum fat-soluble vitamin profiles of the K-means clusters.**

| **Characteristic** | **C1 (n = 2,347)** | **C2 (n = 1,701)** | **C3 (n = 63)** |
| --- | --- | --- | --- |
| Vitamin A | 325.8 (283.6-373.0) | 404.4 (357.8-458.1) | 374.2 (327.6-416.2) |
| 25(OH)D_2_ | 1.0 (0.5-1.7) | 1.0 (0.5-1.8) | 20.4 (15.9-26.5) |
| 25(OH)D_3_ | 19.5 (15.1-24.4) | 29.0 (23.7-34.5) | 14.8 (9.9-21.1) |
| Total 25(OH)D (derived) | 21.0 (16.4-25.9) | 30.3 (25.2-35.8) | 38.6 (29.7-45.7) |
| Vitamin E | 8005.8 (6973.5-9160.8) | 10676.7 (9220.2-12378.4) | 9315.4 (8208.4-12003.3) |
| D2-supplementation marker, % | 7.7 | 3.1 | 100.0 |

*Median (IQR), ng/mL, except the bottom row. Clustered analytes: vitamin A, 25(OH)D_2_, 25(OH)D_3_, vitamin E; total 25(OH)D shown as a derived quantity (not used for clustering). Clusters labeled by ascending mean total 25(OH)D.*

**Table S10. Distribution of K-means clusters across phenotype groups.**

| **Phenotype group** | **C1** | **C2** | **C3** | **Total** |
| --- | --- | --- | --- | --- |
| Control | 786 (52.3%) | 699 (46.5%) | 18 (1.2%) | 1,503 |
| CPP | 798 (69.6%) | 340 (29.7%) | 8 (0.7%) | 1,146 |
| Short stature | 482 (49.7%) | 452 (46.6%) | 36 (3.7%) | 970 |
| Obesity | 281 (57.1%) | 210 (42.7%) | 1 (0.2%) | 492 |

*Counts (within-phenotype row %). Distribution differed across groups (chi-square(6) = 143.1, P < 0.0001).*

**Table S11. Selection of the number of clusters.**

| **Number of clusters (k)** | **Total within-cluster sum of squares** | **Mean silhouette width** |
| --- | --- | --- |
| 2 | 13,003.9 | 0.240 |
| 3 (selected) | 10,363.9 | 0.254 |
| 4 | 8,730.3 | 0.230 |
| 5 | 7,561.7 | 0.233 |
| 6 | 6,881.4 | 0.226 |

*k = 3 selected by maximum mean silhouette width; low absolute values (≈0.25) indicate weak global separation, consistent with the exploratory framing. K-means is stochastic; cluster sizes may differ trivially between software/seeds.*

**Table S12. Internal cross-validation of the primary fat-soluble vitamin profiles (girls 3-13 y, N = 4,111).**

**Panel A. Bootstrap stability of age-adjusted Cohen's d (B = 2,000)**

| **Phenotype** | **Vitamin** | **Original d** | **Boot mean d** | **Boot 95% CI** | **Bias** | **Fold sign consistency** |
| --- | --- | --- | --- | --- | --- | --- |
| CPP | Vitamin A | +0.06 | +0.06 | (-0.02, +0.13) | +0.001 | 7/10 |
| Short stature | Vitamin A | +0.03 | +0.03 | (-0.05, +0.11) | +0.001 | 6/10 |
| Obesity | Vitamin A | +0.50 | +0.50 | (+0.39, +0.61) | +0.001 | 10/10 |
| CPP | 25(OH)D (total) | -0.26 | -0.26 | (-0.33, -0.20) | -0.000 | 10/10 |
| Short stature | 25(OH)D (total) | +0.26 | +0.26 | (+0.19, +0.33) | -0.000 | 10/10 |
| Obesity | 25(OH)D (total) | -0.16 | -0.16 | (-0.23, -0.08) | +0.001 | 8/10 |
| CPP | Vitamin E | -0.25 | -0.26 | (-0.33, -0.18) | -0.000 | 10/10 |
| Short stature | Vitamin E | +0.03 | +0.03 | (-0.04, +0.11) | -0.001 | 4/10 |
| Obesity | Vitamin E | -0.08 | -0.08 | (-0.18, +0.03) | -0.001 | 7/10 |

**Panel B. Bootstrap stability of vitamin D deficiency odds ratios (B = 2,000)**

| **Phenotype vs control** | **Original OR** | **Boot 95% CI** | **Significant (CI excludes 1)** |
| --- | --- | --- | --- |
| CPP | 1.43 | (1.20, 1.72) | Yes |
| Short stature | 0.60 | (0.48, 0.74) | Yes |
| Obesity | 1.23 | (0.97, 1.55) | No |

**Panel C. 10x10 repeated stratified cross-validation: discrimination beyond age (age-only vs age + vitamins)**

| **Phenotype vs control** | **N** | **Age-only AUC** | **Age + vitamins AUC (95% range)** | **Incremental ΔAUC (95% range)** | **Mean Brier** |
| --- | --- | --- | --- | --- | --- |
| CPP | 2,649 | 0.671 | 0.701 (0.650, 0.761) | +0.030 (-0.008, +0.071) | 0.217 |
| Short stature | 2,473 | 0.539 | 0.583 (0.516, 0.653) | +0.044 (-0.035, +0.115) | 0.233 |
| Obesity | 1,995 | 0.717 | 0.765 (0.696, 0.824) | +0.048 (-0.002, +0.090) | 0.159 |

**Panel D. Leave-one-season-out cross-validation: seasonal stability**

| **Phenotype** | **Vitamin** | **Full d** | **Min across seasons** | **Max across seasons** | **Stable** |
| --- | --- | --- | --- | --- | --- |
| CPP | Vitamin A | +0.06 | +0.02 | +0.12 | Yes |
| Short stature | Vitamin A | +0.03 | +0.00 | +0.05 | No (near 0) |
| Obesity | Vitamin A | +0.50 | +0.42 | +0.55 | Yes |
| CPP | 25(OH)D (total) | -0.26 | -0.29 | -0.24 | Yes |
| Short stature | 25(OH)D (total) | +0.26 | +0.23 | +0.29 | Yes |
| Obesity | 25(OH)D (total) | -0.16 | -0.21 | -0.14 | Yes |
| CPP | Vitamin E | -0.25 | -0.29 | -0.22 | Yes |
| Short stature | Vitamin E | +0.03 | -0.05 | +0.10 | No (near 0) |
| Obesity | Vitamin E | -0.08 | -0.09 | -0.05 | Yes |

**Panel E. 10-fold cross-validation of vitamin D-CPP nonlinearity**

| **Model** | **Mean AUC** | **SD** | **dAUC vs linear** | **Paired t** | **P value** |
| --- | --- | --- | --- | --- | --- |
| Linear (age + VD) | 0.688 | 0.039 | - | - | - |
| Polynomial (age + VD + VD^2 + VD^3) | 0.678 | 0.035 | -0.010 | -4.73 | 0.0011 |

*Post-hoc internal validation on the primary cohort (N = 4,111; same as Table 1/2). Bootstrap B = 2,000; fixed seed. 'Original d'/'Original OR' equal Table 2. Panel C: logistic regression (age + 3 vitamins), 10x10 repeated stratified CV. Panel E: polynomial expansion did not improve out-of-sample AUC, so the nonlinearity is primarily threshold-like. Bootstrap/CV are stochastic and vary at the 3rd decimal by seed/software. Panel C additionally reports an age-only model and the incremental ΔAUC (age + three vitamins minus age only), paired within each cross-validation fold; because the phenotype groups differ in age, this isolates the discrimination contributed by the vitamins beyond age. ΔAUC ranges are the 2.5–97.5th percentiles across the 100 folds (folds are correlated, so the range is descriptive).*

**Table S13. Sensitivity of the short-stature 25(OH)D finding to D_2_ (ergocalciferol) supplementation (girls 3-13 y).**

**Panel A. Prevalence of the D_2_-supplementation marker (serum 25(OH)D_2_:D_3_ > 0.2) by phenotype**

| **Group** | **n with marker / N** | **%** |
| --- | --- | --- |
| Control | 92 / 1,503 | 6.1 |
| CPP | 67 / 1,146 | 5.8 |
| Short stature | 117 / 970 | 12.1 |
| Obesity | 19 / 492 | 3.9 |

**Panel B. Age-adjusted standardized difference vs control (Cohen's d, 95% CI), total 25(OH)D decomposed**

| **Component** | **CPP** | **Short stature** | **Obesity** |
| --- | --- | --- | --- |
| Total 25(OH)D | -0.26 (-0.33, -0.20) | +0.26 (0.19, 0.33) | -0.16 (-0.25, -0.07) |
| 25(OH)D_3_ | -0.26 (-0.33, -0.19) | +0.16 (0.09, 0.23) | -0.15 (-0.24, -0.05) |
| 25(OH)D_2_ (supplement-derived) | -0.03 (-0.10, 0.05) | +0.30 (0.22, 0.38) | -0.04 (-0.14, 0.06) |

**Panel C. Robustness of the short-stature 25(OH)D advantage to excluding D2-supplemented children**

| **Measure (short stature vs control)** | **Full cohort (N = 4,111)** | **Excluding D2-supplemented (N = 3,816)** |
| --- | --- | --- |
| Adjusted d, total 25(OH)D | +0.26 (0.19, 0.33) | +0.24 (0.17, 0.32) |
| Adjusted d, 25(OH)D_3_ | +0.16 (0.09, 0.23) | +0.22 (0.15, 0.30) |
| Deficiency OR, total 25(OH)D <=20 | 0.60 (0.48-0.74) | 0.64 (0.51-0.80) |
| Deficiency OR, 25(OH)D_3_ <=20† | 0.72 (0.60-0.88) | — |

*Girls 3-13 y. The D_2_-supplementation marker is serum 25(OH)D_2_:D_3_ > 0.2; its prevalence differed across groups (chi-square P < 0.0001) and was about 2.0 times as high in short stature as in controls (12.1% vs 6.1%, P < 0.0001). Panel B: age-adjusted standardized difference (Cohen's d) versus healthy control, with total 25(OH)D decomposed into 25(OH)D_3_ and ergocalciferol-derived 25(OH)D_2_. Because cutaneous synthesis and oral cholecalciferol (D_3_) supplements both yield 25(OH)D_3_, this component cannot distinguish endogenous from exogenous (supplemental) D_3_. Panel C: the higher 25(OH)D of children with short stature persisted after excluding D2-supplemented children (adjusted d +0.26 -> +0.24) and was also present for 25(OH)D3 (deficiency OR 0.72 vs 0.60 for total 25(OH)D). Because the 25(OH)D_2_:D_3_ ratio is blind to cholecalciferol (D_3_) supplementation, and cholecalciferol is the predominant supplemental form prescribed in our setting, this residual 25(OH)D_3_ difference cannot be read as endogenous; differential supplementation therefore remains a plausible and potentially important contributor to the higher 25(OH)D observed in short stature, although the available data cannot distinguish supplementation effects from phenotype-related or other unmeasured influences. Deficiency is defined as 25(OH)D <=20 ng/mL; †the 25(OH)D_3_ row applies the same threshold to 25(OH)D_3_ alone (an exclusion column is not applicable because removing supplemented children makes D_3_ ~ total). Cohen's d CIs are t-based; odds-ratio CIs are profile-likelihood. All models adjusted for age.*

**Table S14. Sensitivity of primary effect sizes to non-linear (restricted cubic spline) age adjustment (girls 3-13 y, N = 4,111).**

**Panel A. Adjusted Cohen's d versus healthy control: linear vs restricted-cubic-spline age adjustment**

| **Vitamin** | **Phenotype** | **Linear age: d (95% CI)** | **RCS age: d (95% CI)** | **Δd** |
| --- | --- | --- | --- | --- |
| Vitamin A | CPP | +0.06 (-0.02, 0.13) ns | +0.23 (0.14, 0.31) **** | +0.169 |
| Vitamin A | Short stature | +0.03 (-0.05, 0.11) ns | +0.07 (-0.01, 0.15) ns | +0.042 |
| Vitamin A | Obesity | +0.50 (0.39, 0.60) **** | +0.59 (0.49, 0.69) **** | +0.093 |
| 25(OH)D (total) | CPP | -0.26 (-0.33, -0.20) **** | -0.18 (-0.25, -0.11) **** | +0.087 |
| 25(OH)D (total) | Short stature | +0.26 (0.19, 0.33) **** | +0.29 (0.23, 0.36) **** | +0.035 |
| 25(OH)D (total) | Obesity | -0.16 (-0.25, -0.07) ** | -0.11 (-0.20, -0.02) * | +0.048 |
| Vitamin E | CPP | -0.25 (-0.33, -0.18) **** | -0.19 (-0.27, -0.11) **** | +0.064 |
| Vitamin E | Short stature | +0.03 (-0.04, 0.11) ns | +0.06 (-0.02, 0.14) ns | +0.024 |
| Vitamin E | Obesity | -0.08 (-0.18, 0.03) ns | -0.04 (-0.14, 0.06) ns | +0.035 |

**Panel B. Vitamin D deficiency odds ratio versus healthy control: linear vs restricted-cubic-spline age adjustment**

| **Phenotype** | **Linear age: OR (95% CI)** | **RCS age: OR (95% CI)** | **ΔOR (%)** |
| --- | --- | --- | --- |
| CPP | 1.43 (1.20–1.70) | 1.26 (1.04–1.51) | -11.9% |
| Short stature | 0.60 (0.48–0.74) | 0.59 (0.47–0.73) | -1.7% |
| Obesity | 1.23 (0.98–1.55) | 1.15 (0.91–1.44) | -6.7% |

*Sensitivity analysis replacing the linear age covariate with a restricted cubic spline (4 knots at the 5th, 35th, 65th, and 95th percentiles of age; rms package). Panel A: age-adjusted standardized mean differences (Cohen's d) versus healthy controls for all three vitamins and three phenotypes, comparing the primary linear model with the spline model. Panel B: age-adjusted odds ratios of vitamin D deficiency (25(OH)D <=20 ng/mL). FDR-adjusted significance (Benjamini-Hochberg) indicated as in Table 2 (**** P<0.0001, *** P<0.001, ** P<0.01, * P<0.05; ns, not significant). The five principal direction-specific findings (higher vitamin A in obesity, lower 25(OH)D and lower vitamin E in CPP, higher 25(OH)D in short stature) retained their direction and statistical significance under spline-based age adjustment. The CPP-vitamin A association, classified as a borderline finding in the primary analysis, was larger and reached significance under the spline model (d +0.06 to +0.23), consistent with incomplete control of the non-linear age-retinol relationship by a linear term; this reinforces its interpretation as a preliminary finding requiring external replication.*

**Table S15. Sensitivity of the vitamin D_2_ (ergocalciferol) supplementation analysis to the choice of 25(OH)D_2_:25(OH)D_3_ threshold (girls 3–13 y, N = 4,111).**

**Panel A. Prevalence of the D_2_-supplementation marker at each threshold, by phenotype group**

| **25(OH)D_2_:D_3_ threshold** | **Control, n/N (%)** | **CPP, n/N (%)** | **Short stature, n/N (%)** | **Obesity, n/N (%)** |
| --- | --- | --- | --- | --- |
| > 0.10 | 257/1503 (17.1) | 240/1146 (20.9) | 243/970 (25.1) | 94/492 (19.1) |
| > 0.15 | 144/1503 (9.6) | 115/1146 (10.0) | 150/970 (15.5) | 33/492 (6.7) |
| > 0.20 | 92/1503 (6.1) | 67/1146 (5.8) | 117/970 (12.1) | 19/492 (3.9) |
| > 0.30 | 56/1503 (3.7) | 30/1146 (2.6) | 78/970 (8.0) | 8/492 (1.6) |

*Chi-square P < 0.0001 for the between-group difference at every threshold; the short-stature group has the highest prevalence throughout.*

**Panel B. Estimates in marker-negative girls, by threshold**

| **Threshold** | **n retained** | **Short stature, 25(OH)D d** | **Short stature, deficiency OR** | **CPP, 25(OH)D d** | **CPP, deficiency OR** |
| --- | --- | --- | --- | --- | --- |
| > 0.10 | 3,277 | +0.25 (+0.17, +0.33)**** | 0.57 (0.44–0.73)**** | −0.26 (−0.34, −0.19)**** | 1.39 (1.13–1.70)** |
| > 0.15 | 3,669 | +0.25 (+0.17, +0.32)**** | 0.62 (0.49–0.78)**** | −0.27 (−0.34, −0.20)**** | 1.38 (1.15–1.67)*** |
| > 0.20 | 3,816 | +0.24 (+0.17, +0.32)**** | 0.64 (0.51–0.80)**** | −0.28 (−0.35, −0.21)**** | 1.44 (1.20–1.73)**** |
| > 0.30 | 3,939 | +0.24 (+0.17, +0.32)**** | 0.62 (0.50–0.78)**** | −0.28 (−0.34, −0.21)**** | 1.45 (1.21–1.74)**** |

*All estimates are age-adjusted and expressed versus healthy controls, restricted to girls whose 25(OH)D_2_:25(OH)D_3_ ratio was at or below the stated threshold. Adjusted Cohen’s d is the group coefficient from a linear model fitted to total 25(OH)D standardized to unit SD; odds ratios are for vitamin D deficiency (total 25(OH)D ≤ 20 ng/mL) with profile-likelihood 95% CIs. ****P < 0.0001, ***P < 0.001, **P < 0.01.*

*The 0.2 ratio used in the main analysis is a pragmatic, study-defined threshold rather than a validated diagnostic cut-off: 25(OH)D_2_ is normally a negligible fraction of total 25(OH)D, so a ratio above 0.2 indicates a disproportionate ergocalciferol contribution consistent with exogenous D_2_ intake. Moving the threshold between 0.10 and 0.30 changes the proportion of girls classified as supplemented roughly three-fold in every group, yet leaves the short-stature 25(OH)D elevation (d +0.24 to +0.25) and the CPP deficit (d −0.26 to −0.28) essentially unchanged. The conclusions drawn from this marker therefore do not depend on the threshold chosen.*

**Table S16. Sensitivity of the primary phenotype contrasts to continuous adjustment for measured adiposity (girls 3–13 y, N = 4,111).**

**Panel A. Availability of measured height and weight, by phenotype group**

| **Group** | **n analyzed** | **Weight recorded, n (%)** | **Height recorded, n (%)** | **BMI computable, n (%)** |
| --- | --- | --- | --- | --- |
| Healthy control | 1,503 | 1,166 (77.6) | 317 (21.1) | 315 (21.0) |
| CPP | 1,146 | 1,058 (92.3) | 319 (27.8) | 315 (27.5) |
| Short stature | 970 | 858 (88.5) | 228 (23.5) | 223 (23.0) |
| Obesity | 492 | 466 (94.7) | 128 (26.0) | 127 (25.8) |
| *Total* | *4,111* | *3,548 (86.3)* | *992 (24.1)* | *980 (23.8)* |

**Panel B. Measured anthropometry by phenotype group, complete-anthropometry subgroup (n = 980)**

| **Group** | **n** | **Age, y** | **Height, cm** | **Weight, kg** | **BMI, kg/m²** | **BMI-for-age Z** |
| --- | --- | --- | --- | --- | --- | --- |
| Healthy control | 315 | 7.9 (6.0–10.1) | 129.0 (117.8–142.4) | 26.0 (20.6–33.4) | 15.7 (14.5–17.7) | +0.03 (−0.58, +0.89) |
| CPP | 315 | 7.9 (7.2–8.7) | 130.6 (125.7–138.1) | 28.5 (24.5–34.3) | 16.8 (15.3–18.5) | +0.80 (+0.06, +1.66) |
| Short stature | 223 | 6.2 (4.8–8.9) | 108.2 (99.0–122.2) | 17.0 (14.4–22.0) | 14.7 (13.8–15.8) | −0.42 (−1.06, +0.34) |
| Obesity | 127 | 8.8 (7.7–9.9) | 136.2 (128.3–143.6) | 41.1 (34.3–48.0) | 21.9 (20.4–23.6) | +2.52 (+2.08, +3.10) |

*Median (IQR). Kruskal–Wallis P < 0.0001 for height, for BMI and for BMI-for-age Z. Measured anthropometry is concordant with the record-based group labels. Against the same national reference used throughout (Li H et al., refs 29 and 40; L, M and S parameters as tabulated by Zong & Li, ref 41), median height-for-age Z among the 992 girls with a recorded height was −2.21 (−2.46, −1.94) in short stature versus +0.19 (−0.52, +0.83) in controls (Mann–Whitney P < 0.0001), and 68.0% of the short-stature group met the −2 SD diagnostic threshold stated in the Methods (82.0% were at or below the 3rd centile) versus 1.3% of controls. 91.3% of the obesity group were at or above the 95th centile of the national BMI-for-age reference, versus 10.8% of controls and 3.1% of the short-stature group. As stated in the Methods, group assignment reflects the diagnosis recorded by the treating clinician rather than our re-application of the threshold to these measurements. Median weight-for-age Z was +0.02 (control), +0.96 (CPP), −1.71 (short stature) and +2.23 (obesity) in the weight-based set (n = 3,538). In the complete-anthropometry subgroup, weight-for-age Z and BMI-for-age Z were strongly correlated (Pearson r = 0.87).*

**Panel C. Internal validity of the anthropometry subsets: are the phenotype contrasts preserved where adiposity could be measured?**

**C1. Age-adjusted contrasts versus healthy control, estimated separately within each data-availability stratum**

| **Stratum** | **n** | **CPP, 25(OH)D d** | **Short stature, 25(OH)D d** | **CPP, vitamin E d** | **CPP, deficiency OR** | **Short stature, deficiency OR** |
| --- | --- | --- | --- | --- | --- | --- |
| *Full cohort* | *4,111* | *−0.26 (−0.33, −0.20)***** | *+0.26 (+0.19, +0.33)***** | *−0.25 (−0.33, −0.18)***** | *1.43 (1.20–1.70)***** | *0.60 (0.48–0.74)***** |
| BMI computable | 980 | −0.20 (−0.34, −0.07)** | +0.34 (+0.19, +0.49)**** | −0.23 (−0.39, −0.08)** | 1.38 (0.98–1.95) ns | 0.46 (0.29–0.73)*** |
| BMI not computable | 3,131 | −0.28 (−0.36, −0.20)**** | +0.23 (+0.15, +0.31)**** | −0.29 (−0.38, −0.20)**** | 1.46 (1.19–1.80)*** | 0.66 (0.51–0.84)*** |
| Weight analyzable | 3,538 | −0.30 (−0.37, −0.23)**** | +0.29 (+0.22, +0.37)**** | −0.26 (−0.34, −0.18)**** | 1.61 (1.33–1.95)**** | 0.59 (0.46–0.74)**** |
| Weight not analyzable | 573 | +0.13 (−0.10, +0.36) ns | +0.08 (−0.12, +0.28) ns | +0.16 (−0.09, +0.42) ns | 0.50 (0.27–0.90)* | 0.67 (0.38–1.16) ns |

**C2. Formal test of effect modification by data availability (phenotype × availability interaction)**

| **Interaction term** | **Total 25(OH)D, Δd (95% CI)** | **Vitamin E, Δd (95% CI)** | **Deficiency, ratio of ORs (95% CI)** |
| --- | --- | --- | --- |
| CPP × BMI computable | +0.08 (−0.07, +0.24), P = 0.30 | +0.04 (−0.13, +0.22), P = 0.65 | 0.94 (0.63–1.40), P = 0.75 |
| Short stature × BMI computable | +0.08 (−0.09, +0.25), P = 0.37 | +0.15 (−0.04, +0.34), P = 0.12 | 0.72 (0.42–1.21), P = 0.22 |
| CPP × weight analyzable | −0.40 (−0.62, −0.19), P < 0.001 | −0.23 (−0.47, +0.01), P = 0.064 | 3.23 (1.81–5.83), P < 0.001 |
| Short stature × weight analyzable | +0.18 (−0.02, +0.38), P = 0.075 | −0.02 (−0.25, +0.21), P = 0.88 | 0.90 (0.50–1.63), P = 0.73 |

**C3. Relative attenuation on adding continuous adiposity (adjusted estimate ÷ age-only estimate within the same subset)**

| **Measure** | **Phenotype** | **Weight-based set, + weight-for-age Z** | **BMI subgroup, + BMI-for-age Z** |
| --- | --- | --- | --- |
| Vitamin D deficiency, OR | CPP | 0.84 | 0.84 |
|  | Short stature | 1.47 | 1.19 |
| Total 25(OH)D, d | CPP | 0.72 | 0.66 |
|  | Short stature | 0.36 | 0.83 |
| Vitamin E, d | CPP | 0.82 | 0.92 |
|  | Short stature | not interpretable | not interpretable |

*Panel C addresses whether the subsets in which adiposity could be measured are fit for the purpose of asking what adiposity adjustment does. Two forms of unrepresentativeness must be distinguished: unrepresentativeness in the level of a vitamin, which biases prevalence estimates, and unrepresentativeness in the between-group contrast, which would bias the adjustment question itself. Girls with computable BMI differ from those without in vitamin levels (vitamin E higher, vitamin A and total 25(OH)D lower; standardized differences 0.19–0.31, all P < 0.001), but not in the phenotype contrasts: every phenotype × BMI-availability interaction in C2 is null (P ≥ 0.12), and the age-only contrasts estimated inside the BMI subgroup reproduce those of the full cohort (C1). Selection into this subgroup therefore acts on levels rather than on effects, and the subgroup is an internally valid place to estimate the effect of adjustment.*

*The weight-based set behaves differently and is reported here for transparency. The CPP × weight-analyzable interaction is significant (Δd = −0.40, P < 0.001; ratio of ORs 3.23, P < 0.001) and persists under spline age adjustment and after restriction to 5–13 years, so it is not an age-composition artifact. Weight was missing preferentially in 2022–2023 (71% of girls without an analyzable weight versus 39% of those with one) and, among controls, in younger children (median 4.5 versus 7.0 years); within the 573 girls without an analyzable weight the phenotype contrasts are unstable and poorly identified. The practical consequence is that the weight-based set begins from a higher CPP deficiency odds ratio than the full cohort (1.61 versus 1.43), part of which reflects selection rather than adiposity. This is why an age-only model fitted in the identical subset accompanies every adjusted model in Panels D–I, and why the relative attenuation in C3 — not the absolute adjusted value — is the quantity that transports.*

*C3 shows that the two subsets, which differ in size, composition and adiposity measure, agree closely on how much adjustment attenuates the CPP associations (deficiency OR ratio 0.84 versus 0.84; 25(OH)D 0.72 versus 0.66; vitamin E 0.82 versus 0.92). They diverge for short stature on 25(OH)D (0.36 versus 0.83), which is the divergence predicted by the over-adjustment argument set out in the footnotes: weight-for-age partly encodes the short-stature phenotype whereas height-normalized BMI-for-age does not. The vitamin E ratio for short stature is not interpretable because the unadjusted effect is approximately zero.*

**Panel D. Odds of vitamin D deficiency [25(OH)D ≤ 20 ng/mL] versus healthy control**

| **Model** | **CPP vs control** | **Short stature vs control** | **Obesity vs control†** |
| --- | --- | --- | --- |
| ***Weight-based adjustment set (n = 3,538)*** | | | |
| Age only (identical subset) | 1.61 (1.33–1.95)**** | 0.59 (0.46–0.74)**** | 1.30 (1.02–1.65)* |
| + weight-for-age Z | 1.36 (1.11–1.66)** | 0.87 (0.66–1.14) ns | 0.83 (0.63–1.10) ns |
| + season + weight-for-age Z | 1.36 (1.10–1.68)** | 0.74 (0.55–0.98)* | 0.90 (0.66–1.21) ns |
| ***Complete-anthropometry subgroup (n = 980)*** | | | |
| Age only (identical subgroup) | 1.38 (0.98–1.95) ns | 0.46 (0.29–0.73)*** | 1.47 (0.94–2.29) ns |
| + BMI, measured (continuous) | 1.25 (0.88–1.79) ns | 0.52 (0.32–0.82)** | 0.82 (0.47–1.43) ns |
| + BMI-for-age Z | 1.16 (0.80–1.66) ns | 0.55 (0.34–0.87)* | 0.77 (0.45–1.32) ns |
| + season + BMI-for-age Z | 1.11 (0.74–1.65) ns | 0.38 (0.22–0.63)*** | 0.86 (0.48–1.55) ns |
| *Primary model, full cohort (N = 4,111; age-adjusted)* | *1.43 (1.20–1.70)***** | *0.60 (0.48–0.74)***** | *1.23 (0.98–1.55) ns* |

**Panel E. Adjusted Cohen’s d for total 25(OH)D versus healthy control**

| **Model** | **CPP vs control** | **Short stature vs control** | **Obesity vs control†** |
| --- | --- | --- | --- |
| ***Weight-based adjustment set (n = 3,538)*** | | | |
| Age only (identical subset) | −0.30 (−0.37, −0.23)**** | +0.29 (+0.22, +0.37)**** | −0.16 (−0.26, −0.07)*** |
| + weight-for-age Z | −0.22 (−0.29, −0.14)**** | +0.10 (+0.02, +0.19)* | +0.06 (−0.05, +0.17) ns |
| + season + weight-for-age Z | −0.21 (−0.28, −0.14)**** | +0.15 (+0.06, +0.23)*** | +0.02 (−0.08, +0.12) ns |
| ***Complete-anthropometry subgroup (n = 980)*** | | | |
| Age only (identical subgroup) | −0.20 (−0.34, −0.07)** | +0.34 (+0.19, +0.49)**** | −0.17 (−0.35, +0.01) ns |
| + BMI, measured (continuous) | −0.18 (−0.31, −0.04)** | +0.31 (+0.16, +0.46)**** | +0.01 (−0.21, +0.22) ns |
| + BMI-for-age Z | −0.13 (−0.27, +0.00) ns | +0.28 (+0.13, +0.43)*** | +0.08 (−0.13, +0.29) ns |
| + season + BMI-for-age Z | −0.12 (−0.25, +0.01) ns | +0.35 (+0.20, +0.49)**** | +0.04 (−0.16, +0.23) ns |
| *Primary model, full cohort (N = 4,111; age-adjusted)* | *−0.26 (−0.33, −0.20)***** | *+0.26 (+0.19, +0.33)***** | *−0.16 (−0.25, −0.07)**** |

**Panel F. Adjusted Cohen’s d for vitamin E versus healthy control**

| **Model** | **CPP vs control** | **Short stature vs control** | **Obesity vs control†** |
| --- | --- | --- | --- |
| ***Weight-based adjustment set (n = 3,538)*** | | | |
| Age only (identical subset) | −0.26 (−0.34, −0.18)**** | +0.06 (−0.03, +0.15) ns | −0.05 (−0.16, +0.05) ns |
| + weight-for-age Z | −0.21 (−0.30, −0.13)**** | −0.04 (−0.15, +0.06) ns | +0.07 (−0.06, +0.19) ns |
| + season + weight-for-age Z | −0.20 (−0.28, −0.11)**** | −0.04 (−0.14, +0.07) ns | +0.07 (−0.06, +0.19) ns |
| ***Complete-anthropometry subgroup (n = 980)*** | | | |
| Age only (identical subgroup) | −0.23 (−0.39, −0.08)** | +0.15 (−0.02, +0.32) ns | +0.00 (−0.20, +0.20) ns |
| + BMI, measured (continuous) | −0.23 (−0.38, −0.08)** | +0.14 (−0.03, +0.32) ns | +0.04 (−0.21, +0.29) ns |
| + BMI-for-age Z | −0.22 (−0.37, −0.06)** | +0.14 (−0.04, +0.31) ns | +0.06 (−0.18, +0.31) ns |
| + season + BMI-for-age Z | −0.20 (−0.36, −0.05)* | +0.17 (+0.00, +0.34)* | +0.03 (−0.21, +0.27) ns |
| *Primary model, full cohort (N = 4,111; age-adjusted)* | *−0.25 (−0.33, −0.18)***** | *+0.03 (−0.04, +0.11) ns* | *−0.08 (−0.18, +0.03) ns* |

**Panel G. Adjusted Cohen’s d for vitamin A versus healthy control**

| **Model** | **CPP vs control** | **Short stature vs control** | **Obesity vs control†** |
| --- | --- | --- | --- |
| ***Weight-based adjustment set (n = 3,538)*** | | | |
| Age only (identical subset) | +0.02 (−0.07, +0.10) ns | +0.04 (−0.05, +0.12) ns | +0.49 (+0.38, +0.60)**** |
| + weight-for-age Z | −0.09 (−0.17, −0.00)* | +0.26 (+0.16, +0.37)**** | +0.23 (+0.10, +0.35)*** |
| + season + weight-for-age Z | −0.07 (−0.16, +0.01) ns | +0.28 (+0.17, +0.38)**** | +0.22 (+0.09, +0.34)*** |
| ***Complete-anthropometry subgroup (n = 980)*** | | | |
| Age only (identical subgroup) | −0.01 (−0.17, +0.14) ns | +0.07 (−0.10, +0.24) ns | +0.35 (+0.14, +0.55)*** |
| + BMI, measured (continuous) | −0.07 (−0.23, +0.08) ns | +0.14 (−0.03, +0.31) ns | −0.06 (−0.31, +0.19) ns |
| + BMI-for-age Z | −0.09 (−0.25, +0.06) ns | +0.14 (−0.04, +0.31) ns | +0.06 (−0.18, +0.31) ns |
| + season + BMI-for-age Z | −0.08 (−0.24, +0.08) ns | +0.15 (−0.02, +0.33) ns | +0.06 (−0.18, +0.30) ns |
| *Primary model, full cohort (N = 4,111; age-adjusted)* | *+0.06 (−0.02, +0.13) ns* | *+0.03 (−0.05, +0.11) ns* | *+0.50 (+0.39, +0.60)***** |

**Panel H. Direct CPP versus short-stature contrast, with continuous adiposity adjustment**

| **Model** | **n** | **Deficiency OR (95% CI)** | **25(OH)D, d (95% CI)** | **Vitamin E, d (95% CI)** |
| --- | --- | --- | --- | --- |
| ***Weight-based adjustment set*** | | | | |
| Age only (identical subset) | 1,911 | 2.74 (2.19–3.45)**** | −0.58 (−0.66, −0.50)**** | −0.34 (−0.44, −0.25)**** |
| + weight-for-age Z | 1,911 | 1.38 (0.97–1.95) ns | −0.30 (−0.42, −0.17)**** | −0.10 (−0.24, +0.05) ns |
| ***Complete-anthropometry subgroup*** | | | | |
| Age only (identical subgroup) | 538 | 2.96 (1.92–4.64)**** | −0.53 (−0.69, −0.38)**** | −0.37 (−0.54, −0.20)**** |
| + BMI, measured (continuous) | 538 | 2.28 (1.43–3.69)*** | −0.46 (−0.62, −0.29)**** | −0.37 (−0.55, −0.18)**** |
| + BMI-for-age Z | 538 | 2.02 (1.25–3.33)** | −0.39 (−0.57, −0.22)**** | −0.34 (−0.54, −0.15)*** |
| *Primary model, full cohort* | *2,116* | *2.40 (1.95–2.97)***** | *−0.54 (−0.61, −0.46)***** | *−0.33 (−0.42, −0.24)***** |

**Panel I. Association of adiposity itself with each vitamin (age-adjusted), confirming that the confounding pathway is present**

| **Outcome** | **Per +1 BMI-for-age Z (n = 980)** | **Per +1 weight-for-age Z (n = 3,538)** |
| --- | --- | --- |
| Total 25(OH)D, d | −0.129 (−0.168, −0.090)**** | −0.132 (−0.149, −0.114)**** |
| Vitamin A, d | +0.109 (+0.065, +0.154)**** | +0.096 (+0.076, +0.116)**** |
| Vitamin E, d | −0.042 (−0.086, +0.002) ns | −0.060 (−0.080, −0.040)**** |
| Vitamin D deficiency, OR | 1.35 (1.22–1.50)**** | 1.27 (1.21–1.33)**** |

*Height and weight recorded at the testing encounter were retrieved from the electronic medical record for the whole analytic cohort and linked to the laboratory file by specimen barcode; all 4,111 girls were matched. A weight was recorded for 3,548 girls (86.3%). Before analysis, 10 of these were set to missing by a pre-specified plausibility screen: 7 recorded weights exceeded 100 kg in a girl aged 3–13 years, and in every one of those records no height had been entered and the value read as a height in centimeters fell within 1 SD of the median height-for-age, indicating that a height had been entered in the weight field; a further 3 had a weight-for-age Z below −6, the WHO lower limit of biological plausibility. The WHO upper flag (+5 SD) was deliberately not applied, because this cohort over-samples obesity by design and girls above that limit are the phenotype of interest rather than errors. The weight-based analysis set is therefore n = 3,538 (86.1%). None of the excluded records carried a height, so the complete-anthropometry subgroup (n = 980, 23.8%) is unaffected. Retaining all 3,548 recorded weights changes no estimate in Panels D to G by more than 0.03 in d or 0.05 in the odds ratio, and changes no conclusion.*

*Each adiposity-adjusted model is reported together with an age-only model fitted in the identical subset, so that the contribution of adiposity adjustment is separated from the contribution of sample restriction. The complete-anthropometry subgroup is underpowered: with 315 CPP and 315 control girls and a control deficiency prevalence of 34.3%, power to detect the primary OR of 1.43 is 58%, and the age-only model in that same subgroup is already non-significant (OR 1.38, 0.98–1.95).*

*†The obesity column is shown for completeness only and is not interpreted: the obesity group is defined by BMI, so conditioning on continuous BMI removes the exposure itself (over-adjustment). The same caution applies in weaker form to the short-stature group when weight-for-age is used: median weight-for-age Z in short stature was −1.71 whereas median BMI-for-age Z was only −0.42, indicating that the weight deficit is predominantly height (the phenotype) rather than adiposity. The height-normalized BMI-for-age Z estimate is therefore the more appropriate adiposity adjustment for short stature, and it leaves the 25(OH)D elevation essentially intact (+0.34 to +0.28), whereas weight-for-age adjustment attenuates it further (+0.29 to +0.10). After additionally excluding girls positive for the D_2_-supplementation marker, the short-stature 25(OH)D elevation adjusted for BMI-for-age Z was d = +0.25 (+0.09, +0.41).*

*Further attenuation of the CPP deficiency odds under flexible (restricted-cubic-spline) age adjustment is an age effect rather than an adiposity effect and is reported separately in Table S14.*

*BMI-for-age Z, weight-for-age Z and height-for-age Z were computed by the LMS method [Z = ((X/M)^L − 1)/(LS)] against the national growth references for Chinese children and adolescents aged 0–18 years (Li H et al., refs 29 and 40). The L, M and S parameters were taken from the tabulated values published for the identical reference sample (69,760 children under 7 years plus 24,542 school children; n = 94,302) in Zong X-N and Li H, PLoS ONE 2013;8(3):e59569, Tables 2, 3 and 7 (ref 41, open access), and were interpolated to exact age in months with a monotone cubic spline; for ages 3 years and above the standing-height rows were used. The raw continuous BMI models (Panels D to H) require no growth reference at all and gave concordant estimates throughout, indicating that the conclusions do not depend on the choice of reference.*
